# Structural and mechanistic basis for recognition of alternative tRNA precursor substrates by bacterial ribonuclease P

**DOI:** 10.1038/s41467-022-32843-7

**Published:** 2022-08-31

**Authors:** Jiaqiang Zhu, Wei Huang, Jing Zhao, Loc Huynh, Derek J. Taylor, Michael E. Harris

**Affiliations:** 1 Department of Chemistry, University of Florida, Gainesville, FL USA; 2 Department of Pharmacology, Case Western Reserve University School of Medicine, Cleveland, OH USA; 3 Department of Biochemistry, Case Western Reserve University School of Medicine, Cleveland, OH USA

**Keywords:** Cryoelectron microscopy, Catalytic RNA, Enzymes, Non-coding RNAs

## Abstract

Binding of precursor tRNAs (ptRNAs) by bacterial ribonuclease P (RNase P) involves an encounter complex (ES) that isomerizes to a catalytic conformation (ES*). However, the structures of intermediates and the conformational changes that occur during binding are poorly understood. Here, we show that pairing between the 5′ leader and 3′RCCA extending the acceptor stem of ptRNA inhibits ES* formation. Cryo-electron microscopy single particle analysis reveals a dynamic enzyme that becomes ordered upon formation of ES* in which extended acceptor stem pairing is unwound. Comparisons of structures with alternative ptRNAs reveals that once unwinding is completed RNase P primarily uses stacking interactions and shape complementarity to accommodate alternative sequences at its cleavage site. Our study reveals active site interactions and conformational changes that drive molecular recognition by RNase P and lays the foundation for understanding how binding interactions are linked to helix unwinding and catalysis.

## Abstract (web summary)

Ribonuclease P efficiently processes all tRNA precursors despite sequence variation at the site of cleavage. Here, authors use high-throughput enzymology and cryoEM to reveal conformational changes that drive recognition by bacterial RNase P.

## Introduction

**Fig. 1:**
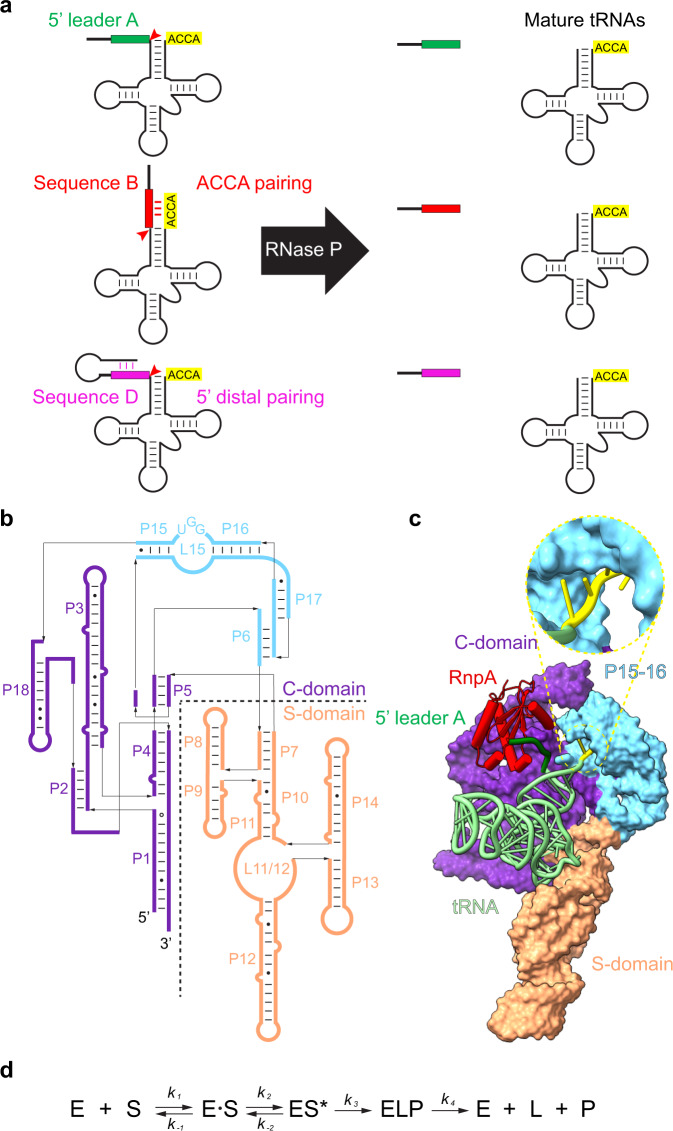
Structure and function of bacterial RNase P. **a** RNase P (black arrow) cleaves ptRNA to remove 5′ leader sequences and generate the mature tRNA (green, red, purple). The sequence of 5′ leaders are variable and are optimally single stranded (Sequence A) but can potentially form pairs with the 3′ RCCA (Sequence B) or fold back to form a stem-loop occluding the 5′ leader (Sequence B). **b** Secondary structure of *T. maritima* RNase P RNA. **c** Structure of *T. maritima* RNase P complex with product tRNA (PDB entry: 3Q1R)^10^. Zoom-in view for the positioning of the 3′ RCCA sequence. P RNA C-domain is colored purple; the S-domain is orange; L15 region is cyan; rnpA is red; tRNA is gray; 5′ leader is green. **d** Steps of RNase P catalyzed enzymatic reaction according to Michaelis-Menten mechanism: a substrate S binds reversibly with the enzyme E to form an enzyme-substrate complex ES, subsequently the conformational change introduces the catalytic active ES* state to enable the proceeding of reaction to enzyme-leaving group-product complex, and enzyme E is finally regenerated for the next catalytic cycle.

Ribonuclease P (RNase P) is an endonuclease whose primary role is to remove diverse 5′ leader sequences from precursor tRNAs (ptRNAs) (Fig. 1a)^1–3^. RNase P functions as a ribonucleoprotein with a conserved RNA subunit (P RNA) and a variable number of protein subunits, although protein RNase P occurs in some eukaryotic nuclei and organelles^4^. Bacterial RNase P consists of a ~400 nucleotide P RNA and a single protein (rnpA, *~*120 amino acids). P RNA contains the active site^5^, while both RNA and protein components contribute to substrate binding^3,6–8^. Together they collaborate to process ptRNAs, and precursors to transfer-messenger RNA (tmRNA), 4.5S rRNA, and many other targets in the transcriptome^1,9^.

X-ray crystal structures of bacterial RNase P and cryoEM structures of archaeal and eukaryotic RNase P bound to ptRNA or tRNA reveal common structures and shared modes of substrate recognition^10–13^. As exemplified by RNase P from *Thermotoga maritima*, P RNA bound to product tRNA is composed of independently folding catalytic domain (C-domain) and specificity domain (S-domain) (Fig. 1b, c). RNase P recognizes the shape of the stacked acceptor and T-stems which span the two domains that together form a molecular ruler^14^. In bacterial RNase P the 3′ RCCA of ptRNA pairs with a loop or internal bulge in the C-domain (L15), while a conserved adenosine in the catalytic core contacts an optimal U(−1) flanking the cleavage site 5′ to N(1)^8,15,16^. The rnpA subunit binds near the P RNA active site and contacts leader nucleotides 5′ to N(−3) to enhance substrate affinity and catalytic metal ion binding^17,18^. In addition to these determinants, RNase P cleavage is negatively impacted by base pairing of proximal ptRNA 5′ leader nucleotides with the 3′ RCCA or by distal leader sequences with nucleotides proximal to the cleavage site (Fig. 1a). Both kinds of structural anti-determinants occur in endogenous substrates^19–24^ and can affect cleavage rate and specificity in vitro and in vivo^25–28^. However, we lack knowledge of the structure of bacterial RNase P bound to substrate ptRNA in a pre-cleavage complex. This gap in our knowledge presents a barrier to achieving a comprehensive description of RNase P specificity and understanding the mechanistic basis for unwinding intramolecular pairing among alternative substrates.

CryoEM structures of human, yeast, and archaeal RNase P show that these enzymes are largely pre-organized for efficient substrate binding^11–13^. However, comparison of free and ptRNA-bound yeast RNase P identified a U in the conserved P4 helix that undergoes rotation upon substrate binding facilitating coordination of a catalytic Mg^2+^ ion^12^. Bacterial RNase P substrate binding appears more dynamic. Stopped flow studies of *B. subtilis* RNase P demonstrate a two-step association mechanism with an association step near the diffusion limit to form a weak encounter complex (ES)^29,30^. This is followed by a conformational change that enhances the overall affinity of ptRNA and is linked to catalytic metal ion binding (ES*)^29–34^. A fundamental unresolved question is, what are the structures and mechanisms that underlie these important functional dynamics in bacterial RNase P and ptRNA that occur during formation of ES*.

Here, we demonstrate that during ptRNA processing by *E. coli* RNase P, the presence of pairing interactions that lengthen the acceptor stem inhibit a conformational change required for catalysis. CryoEM structures of RNase P bound to ptRNA with different leader sequences reveal that these pairing interactions are unwound in the pre-catalytic complex. The data reveal a highly dynamic free RNase P holoenzyme and show how the RNA and protein subunits collaborate to recognize the 5’ leader, position the reactive phosphoryl group for catalysis, and accommodate variation in substrate sequence. Our study reveals active site interactions and conformational changes during molecular recognition by RNase P and provides a framework for understanding their contribution to discrimination between alternative substrates in vivo.

## Results

### Comprehensive identification of ptRNA 5′ leader determinants and anti-determinants

**Fig. 2:**
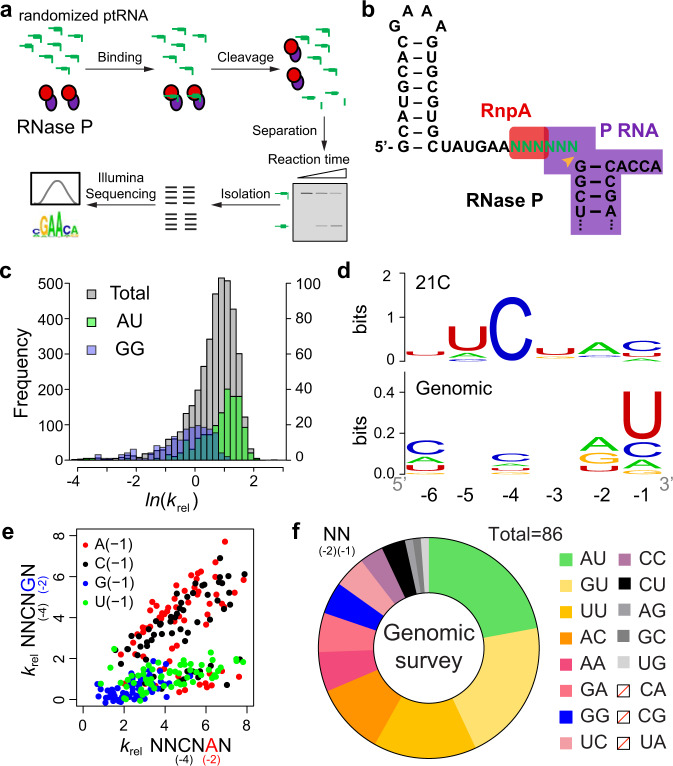
HTS-Kin analysis of RNase P 5’ leader determinants and anti-determinants due to 3’ RCCA pairing. **a** High-throughput sequencing kinetics (HTS-Kin) involves generation of a randomized pool of substrate RNAs, ptRNAs for RNase P, the residual substrate population at different time points are purified and subject to Illumina sequencing. Internal competition kinetics is used to calculate relative rate constants for each sequence from the read data yielding a distribution from which comprehensive specificity determinants can be interpreted. **b** Sequence and 5′ leader structure of the ptRNA^met^(N(−6) to N(−1)) randomized pool. The RNase P cleavage site is indicated by an orange arrow, the randomized positions are green. Red and purple boxes indicate the interactions between the substrate and P RNA, rnpA protein, respectively. **c** Rate constant distribution show as a plot of the number of sequences binned according to their *k*_*rel*_ values calibrated to the genomically encoded leader sequence (*k*_rel_ = (*k*_cat_/*K*_m_(NNNNNN))/(*k*_cat_/*K*_m_(AAAAAG))). y-axis on the left shows the frequencies of total population and y-axis on the right shows frequencies of ptRNA_AU and ptRNA_GG subsets. **d** Optimal sequence logo for the fastest 1% of sequences from the 21C rate constant distribution (top) And the sequence logo calculated from endogenous *E. coli* ptRNA leader sequences (bottom). **e** Dot plot of *k*_rel_ values comparing the effect of an A(−2) to G(−2) mutation in the context of all other sequence combinations. This plot shows only the subset with an optimal C(−4), the plots for other N(−4) nucleotides is included in Supplementary Fig. 3. **f** Distribution of nucleotides of optimal and non-optimal sequences at N(−1)N(−2) for endogenous *E. coli* ptRNAs. Source data are provided as a Source Data file.

The specificity of RNase P for ptRNA is due to favorable interactions with tRNA and the 5′ leader, as well as the inhibitory structure involving nucleotides adjacent to the cleavage site (Fig. 1a)^3,8^. These structural anti-determinants can involve the formation of stem loops in the 5′ leader^19,24^ or pairing between the 3′ RCCA of tRNA and N(−1) to N(−3)^27,35,36^. To comprehensively identify and parse sequence determinants and anti-determinants in the 5′ leader, we used high-throughput sequencing kinetics (HTS-Kin) to measure the relative *k*_cat_/*K*_m_ for all possible combinations of 5′ leader nucleotides N(−6) to N(−1) (*n* = 4096)^19–21,37^. HTS-Kin measures the time-dependent changes in distribution of ptRNA in the population due to RNase P processing by Illumina sequencing (Fig. 2a)^20,38^. Relative rate constants are calibrated to the genomically encoded leader sequence^38–40^. Distal 5′ sequences were added to ptRNA^met^ to facilitate sequencing^37^. To sequester these nucleotides and prevent formation of inhibitory structure observed in previous HTS-Kin experiments^19^, we engineered a stem loop in the 5′ distal sequence in the ptRNA^met^(N(−6) to N(−1))_21C randomized pool used for HTS-Kin (Fig. 2b). The rate constant distributions determined at three different fractions of reaction for two independent technical replicates were highly similar (Supplementary Fig. 1). As expected, the greatest errors are for the slow reacting sequences for which the change in sequence reads over time was minimal^38,40^.

The rate constant distribution determined by HTS-Kin for the ptRNA^met^(N(−6) to N(−1))_21C pool spans a *ca*. 100-fold range and describes the spectrum of effects of randomization on *k*_cat_/*K*_m_ (Fig. 2c). The optimal sequence logo calculated for the fastest 1% of substrate variants shows preference for A at N(−2) and C at N(−4) while U at N(−5) and N(−6) are optimal (Fig. 2d). The results are consistent with previous binding studies showing that both *Bacillus subtilis* and *E. coli* RNase P binding are enhanced by sequence-specific contacts with N(−4)^41^. An A at N(−2) is consistently observed as a positive determinant even in the P RNA alone reaction consistent with an RNA-RNA contact, but the basis for this is not known^21^. A logo comparing endogenous *E. coli* ptRNA leaders shows modest conservation of U at N(−1) which is known to be optimal for the cleavage step, less conservation is observed for A or G at N(−2) and C at N(−4) consistent with the HTS-Kin data reflecting biological specificity.

Previous kinetics and HTS-Kin analysis showed that pairing of the 5′ leader with the 3′ RCCA of ptRNA acts as an anti-determinant for RNase P cleavage^19–21,27,35,36^. However, interference from stem-loop formation with distal 5’ sequences precluded a complete quantitative description of the effects of 3’ RCCA pairing. The large effect of N(−1)N(−2) identity on *k*_cat_ /*K*_m_ ptRNAs observed previously is illustrated by the distribution of *k*_rel_ values for ptRNAs with dinucleotide sequences GG, GU, CG, and UG which had the lowest *k*_rel_ while AU, AA, and AC are optimal. To examine this, we fit the 21C rate constant distribution to a quantitative sequence specificity model that includes both position weight matrix (PWM) scores and interaction terms (IC values) to quantify coupling between positions which can represent structure preferences (Supplementary Fig. 2). The PWM scores confirm the determinants observed in optimal consensus logos and show G is an anti-determinant at N(−1) to N(−4). Analysis of IC values is consistent with dinucleotides GU, GG, and UG that can all form at least 2 pairs with the 3’RCCA have large effects on *k*_cat_/*K*_m_ relative to positive sequence determinants. To test the basis for these effects we compared the *k*_rel_ values for subsets of ptRNA with optimal C(−4) but either A or G at N(−2). The data show that changing A to G at N(−2) results in a large decrease in *k*_cat_/*K*_m_ when N(−1) is either U or G, but not C or A (Fig. 2e). When N(−4) is G then an A to G change at N(−2) results in a decrease in *k*_cat_/*K*_m_ regardless of N(−1) identity (Supplementary Fig. 3). These results confirm A(−2) and C(−4) are positive determinants and reveal a threshold of two pairs between 5′ leader and 3’ RCCA extending the acceptor stem decreases *k*_cat_/*K*_m_. Among the endogenous 87 ptRNA in *E. coli* 18 have G(−2)U(−1) and 5 have G(−2)G(−1), while 15 ptRNA have an optimal A(−2)U(−1) (Fig. 2f). Thus, the accommodation of *E. coli* RNase P with inhibitory pairing interaction involving nucleotides adjacent to the cleavage site is a routine part of its biological function.

### Pairing between ptRNA 5′ leader N(−1)N(−2) and 3′ RCCA inhibits conversion of ES to ES*

To identify how 3′ RCCA pairing exerts its effect on cleavage and how RNase P accommodates this variation in sequence and structure, we measured multiple turnover kinetics, binding affinity, and dissociation kinetics of ptRNA_AU and ptRNA_GG. The ptRNA_AU substrate has the optimal consensus leader while ptRNA_GG contains the slowest dinucleotide sequence at N(−1)N(−2). We measured a 13-fold lower *k*_cat_/*K*_m_ for ptRNA_GG compared to ptRNA_AU in single substrate assays (Table 1). Binding affinity and dissociation kinetics were analysed by EMSA in the presence of Ca^2+^. RNase P-ptRNA complexes formed in Ca^2+^ instead of Mg^2+^ make native interactions with ptRNA, but have ~500-fold slower cleavage^30,42–45^. We observed ptRNA_GG has only a twofold lower *K*_d_ compared to ptRNA_AU despite the >10-fold difference in *k*_cat_/*K*_m_ revealing differences in commitments to catalysis such as effects on a conformational change (Supplementary Fig. 4, Table 1).

**Table 1:** Multiple turnover kinetics, binding affinity, and dissociation kinetics of ptRNA_AU and ptRNA_GG

ptRNA	*k*_cat_ (s^−1^)	*k*_cat_/*K*_m_ (M^−1^ s^−1^ × 10^6^)	*K*_d_ (nM) (3 mM Ca^2+^)
AU	0.12 ± 0.01	7 ± 2	35 ± 5
AU_T5	0.0250 ± 0.0003	1.3 ± 0.1	Nd
AU_D3	0.261 ± 0.009	26 ± 8	Nd
AU_T5D3	0.060 ± 0.003	3.1 ± 0.9	Nd
GG	0.07 ± 0.01	0.5 ± 0.2	63 ± 8
GG_D3	0.048 ± 0.001	4.2 ± 0.9	Nd

**Fig. 3:**
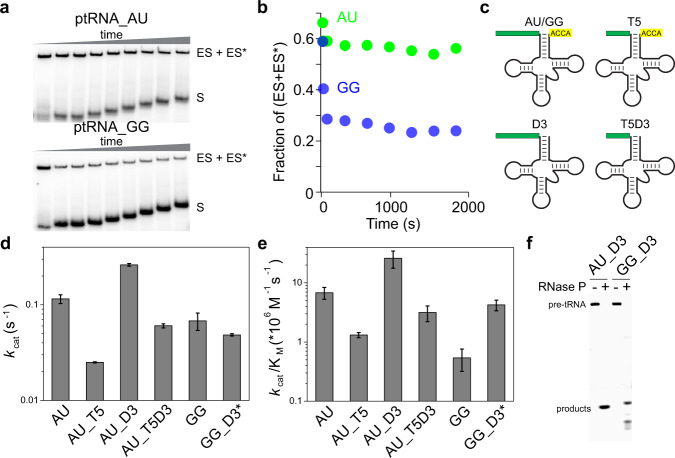
Pairing interactions with 3′ RCCA limit formation of ES*. **a** Analysis of ptRNA_AU and ptRNA_GG dissociation kinetics. 5′ ^32^P-labeled ptRNA_AU (top) and ptRNA_GG (bottom) was bound to 150 nM RNase P in 5 mM Ca^2+^ and then the complexes were challenged with an excess (500 nM) of non-radiolabeled ptRNA_GG. The dissociation of ptRNA from ES/ES* was quantified by native EMSA in three independent experiments with essentially identical results. **b** Quantification of ptRNA remaining in stable ES* complexes fraction. **c** Schematic of different ptRNA substrate constructs used to examine the effect of 5′ leader length and 3′RCCA sequence on enzyme kinetics. T5: truncation of the ptRNA 5′ leader to two nucleotides; D3: 3′ RCCA deletion. The presence of mis-cleavage for the GG_D3 substrate is indicated by an asterisk. **d–f** Cleavage of 5′ ^32^P-labeled AU_D3 and GG_D3. Mis-cleavage of GG_D3 was observed in all reactions used for analysis of kinetic parameters. Two independent experiments specifically analysing cleavage products demonstrated similar results. Reactions without and with RNase P are marked by minus (−) and plus (+) symbols, respectively. Source data are provided as a Source Data file.

Dissociation kinetics of ptRNA_AU and ptRNA_GG complexed with RNase P were measured by adding an excess of unlabeled ptRNA to preformed complexes of labeled substrates and quantifying dissociation using EMSA (Fig. 3a, b). Only a small percentage of ptRNA_AU dissociates during the entire time course. In contrast approximately half of the bound ptRNA_GG dissociated rapidly while the remainder was still bound. This result demonstrates that both substrates form stable complexes with RNase P (ES*) as well as unstable complexes that dissociate rapidly (ES). The fraction of substrates that accumulated in the unstable (ES) complex is greater for the ptRNA_GG consistent with 3′ RCCA pairing inhibiting formation of ES*.

To test the effects of 3′ RCCA and 5′ leader pairing on reaction kinetics, we measured the effect of truncating the 5′ leader to two nucleotides (T5) and deleting 3′ RCCA (D3) on *E. coli* RNase P processing of ptRNA_AU and ptRNA_GG (Fig. 3, Table 1). Truncation of the 5′ leader to two nucleotides reduces *k*_cat_ by 4.6-fold and *k*_cat_/*K*_m_ by 5.2-fold for ptRNA_AU consistent with loss of 5′ leader interaction with rnpA. Deletion of 3′ RCCA results in an increase of 3.8-fold and 7.9-fold in *k*_cat_/*K*_m_ for ptRNA_AU and ptRNA_GG, respectively, while not significantly affecting *k*_cat_. This result is consistent with interaction between the 5′ leader and 3′ RCCA inhibiting the transition from ES to ES*, while not significantly affecting the cleavage step once the substrate is bound in the ES* complex. This increase is more significant for ptRNA_GG where G(−2)G(−1) can form two base pairs with 3′ RCCA. Interestingly, mis-cleavage is observed for ptRNA_GG_D3 at U(−3) and C(−4) (Fig. 3f), due to loss of 3′ RCCA interactions required to position the substrate in the active site in the absence of an optimal interactions with N(−1)^23,31,36^. Together these results provide an explanation for the slower reaction kinetics is likely due to 3′ RCCA pairing with the first two 5′ leader nucleotides by presenting a barrier to formation of the active ES* complex. Once this barrier is crossed the N(−1)N(−2) nucleotides and 3’ RCCA become available for binding to the P RNA active site and L15, respectively.

### Structural basis for substrate accommodation by *E. coli* RNase P

Upon the unpairing of 3′ RCCA, docking into the active site requires accommodation of differences at N(−1) and N(−2) positions. To better understand how unwinding occurs and delineate the structural basis for such plasticity, we used cryo-electron microscopy (cryoEM) single particle analysis (SPA) to determine the structures of holoenzyme and ES* complexes formed with ptRNA_GG and ptRNA_AU. To enrich the ES* state we assembled *E.coli* RNase P holoenzyme with both ptRNA substrates in the presence of Ca^2+^ (see below)^29,42^. Complexes eluting as a peak from size-exclusion chromatography that corresponds to RNase P-ptRNA complex were subjected to high-resolution cryoEM data collection.

**Fig. 4:**
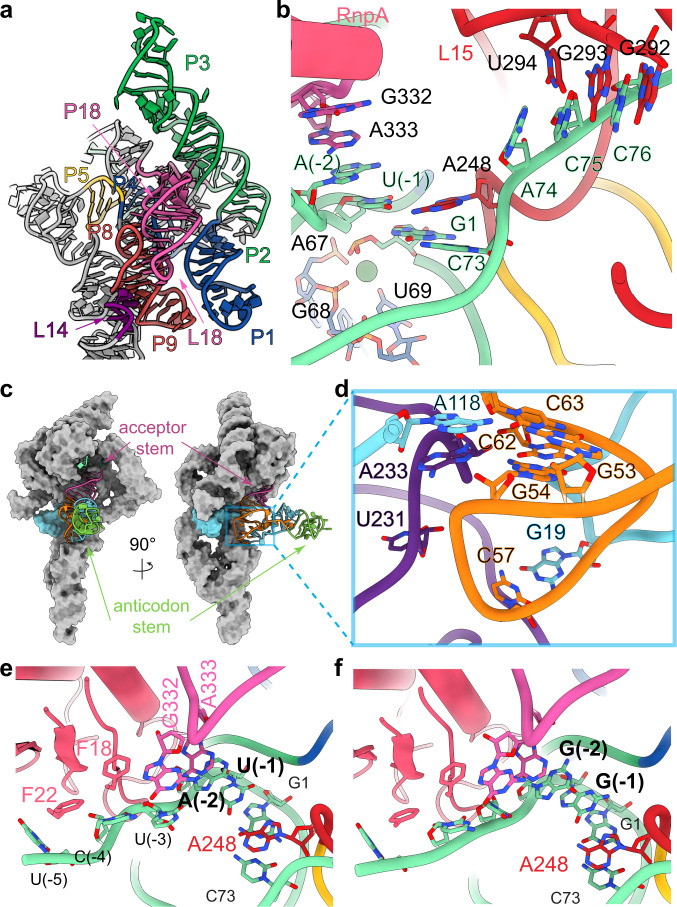
CryoEM structures of ES* complex of ptRNA_AU and ptRNA_GG and E. coli RNase P. **a** Colored structure model highlighting three sets of coaxial stacked helices P2–P3 (green), P1–P4 (blue), P5 (yellow) and P8–P9 (cyan) as well as tetraloop-helix interactions between L14 (purple), L18 (pink) and P8–P9 (red). **b** Overall view of the active site in ptRNA_AU bound *E. coli* RNase P structure showing interactions between P RNA and rnpA (pink) with the ptRNA 5′ leader sequence (green) and base pairings between ptRNA 3′ACC and G292-U294 of L15 internal loop. An inner-sphere Ca^2+^ coordinates phosphate groups of A67, G68, and O4 of U69 of P RNA and the phosphate group of ptRNA G1. **c** Global view of ES* complex showing the binding model of ptRNA (in cartoon representation, acceptor stem: magenta, anticodon stem: green, D-loop: cyan, TΨC loop: orange) to RNase P (in surface representation, P8–P9: cyan). **d** Close-up view of the tertiary interactions between S-domain (purple) and ptRNA (same color codes as **c**). A118 bulge of P9 stacks on A233 and forms base triple with G53–C63 base pair of ptRNA. **e**, **f** Close-up view of 5′-leader sequence interactions with RNase P for AU_ES* (**e**) and GG_ES* (**f**).

The overall architecture of the *E. coli* ptRNA-RNase P complex is similar to that of *T. maritima* RNase P bound to a tRNA product^10^. Three sets of coaxial stacked helices P2–P3, P1–P4–P5, and P8–P9 constitute the conserved core (Fig. 4a). Similarly, the L14 and L18 tetraloops dock into P8, and tetraloop-helix interactions involving L8 and P4, and L9 and P1 connect the C- and S-domains of P RNA (Fig. 4a). The ptRNA anticodon stem extends away from RNase P and the acceptor stem interacts with the P RNA (Fig. 4c). The G19-C56 base pair of ptRNA, a tertiary interaction between D-loop and TѰC-loop of tRNA, stacks into the S-domain T-loops (Fig. 4d). The proximity of U231 to the substrate is consistent with the observation of crosslinking with ptRNA by photocrosslinking^46^. In addition to the sensing of the acceptor arm binding by A233 (A207 in *T. maritima*), the A118 bulge of P9 in *E. coli* P RNA stacks on A233 (Fig. 4d). Therefore, the P8-9 helices play multifaceted roles, not only in connecting the C- and S-domains, but also is likely to communicate the binding of tRNA substrates (Fig. 4c). RnpA adopts the expected αβ sandwich fold and binds to the junction between the P2–P3 and P1–P4–P5 stacked helixes contacting J18/2^2,10,47,48^.

The 5′ end of mature tRNA extends away from the active site at a region where the protein, P RNA, and tRNA converge. In both ptRNA_AU and ptRNA_GG bound complexes, the 5′ leader is positioned with the correct phosphodiester bond placed in the active site, while the 3′ RCCA is trapped in the L15 internal bulge loop of RNase P (Fig. 4b). A comparison of the two structures helps to explain previous biochemical data^10,41,49–51^ as they relate to interactions that exist between the 5′ leader of ptRNA and RNase P. Notably, the N(+1)–N(+73) base pair of ptRNA stacks on a conserved adenosine (A248 in *E. coli*) in J5/15 of RNase P (Fig. 4b). Our structures reveal that the nearly universally conserved A248 nucleotide stacks with the G1-C73 base pair of ptRNA to interact with and properly position N(−1) of the substrate for optimal processing^15,16^. While U at N(−1) is optimal for catalysis, it does not contribute significantly to *k*_cat_/*K*_m_ when catalysis is not rate limiting^16,21^. When a smaller nucleobase occupies the N(−1) position of the leader sequence, as in the case of the ptRNA_AU-bound substrate, it forms a parallel stacking interaction with G1-C73 base pair of ptRNA and A248 of RNase P (Fig. 4e). In contrast, a larger nucleobase at the N(−1) position of ptRNA leader sequence, as in the case of ptRNA_GG-bound complex, sits in the cavity between J5/15 and J18/2 of RNase P thereby forcing the A248 nucleobase to tilt away from the leader sequence and stack with C73 of ptRNA (Fig. 4f). As such, RNase P uses a rotating glycosidic bond of A248 to help sculpt a flexible binding site to accommodate different nucleotides at N(−1), all while maintaining the correct active site geometry for the transition state. The N(−2) position of the ptRNA leader stacks with A333 of RNase P in both the ptRNA_AU and ptRNA_GG complexes (Fig. 4e, f). Not surprisingly, photocrosslinking assays have mapped the 5′ end of tRNA and the cleavage site of ptRNA to be adjacent to A248/249 and A333 that localize to J5/15 and J18/2, respectively, of RNase P^46,52,53^.

Whereas positions N(−1) and N(−2) exhibit well defined features those of the remaining nucleotides in the leader sequence of ptRNA, up to N(−5), are less apparent in the cryoEM map (Supplementary Fig. 5a, b). Despite both complexes sharing a uridine at the same −3 position of the leader sequence, the cryoEM map has stronger features for this residue in the ptRNA_GG complex where the nucleobase is oriented to form a potential interaction with G332 of RNase P. The N4 amine of C(−4) is perfectly positioned to hydrogen-bond with O6 of G332 of RNase P, which would explain the preference of a cytosine at this position of the leader that we observed in the HTS-Kin and equilibrium binding assays (Fig. 2). As such, our structures provide the molecular basis for protection of G332 from kethoxal modification when a ptRNA leader of at least 3-4 nucleotides is bound^45^. It is worth noting that in *B. subtilis* RNase P and other P RNAs, the homologous residue is an adenosine (A318), which could explain the preference for U or A at the N(−4) position for this enzyme^41^. The N(−4) nucleotide also interacts with rnpA via a hydrophobic patch that surrounds F22 of the protein. Mutation of the analogous residue (F17A) in *T. maritima* rnpA decreases enzyme activity by 10-fold, presumably by disrupting the interaction with the leader sequence^50^. At the U(−5), only map features for the phosphodiester backbone are observed in both AU_ES* and GG_ES* cryoEM maps. Altogether, these data are fully consistent with previous biochemical data demonstrating that leader sequences prior to N(−4) have little effect on cleavage kinetics^54^.

The 3′ end of ptRNA is fully extended into the deep pocket formed by the L15 internal loop. Like the *T. maritima* complex bound to product tRNA, 3′ RCCA forms Watson-Crick base pairing with G292-U294, while G291 and A258 form a sheared G-A base pair to kink the helix (Supplementary Fig. 5c). This kink, together with the L16 bulge, stabilizes the pseudoknot between G276-C279 and G82-C85, sculpting an arc-like shape of P15-17 helices. A key difference between two RNase P complexes arises in the protein component. RnpA of *E. coli* RNase P lacks a positive charge surface, presented by R12, R14, and R15 in *T. maritima* RNase P that contacts the phosphate backbone of nucleotides in the L15 internal bulge (Supplementary Fig. 5d). In *T. maritima* RNase P this feature could provide enhanced thermal stability relative to the *E. coli* enzyme.

### Induced fit at the active site of RNase P

**Fig. 5:**
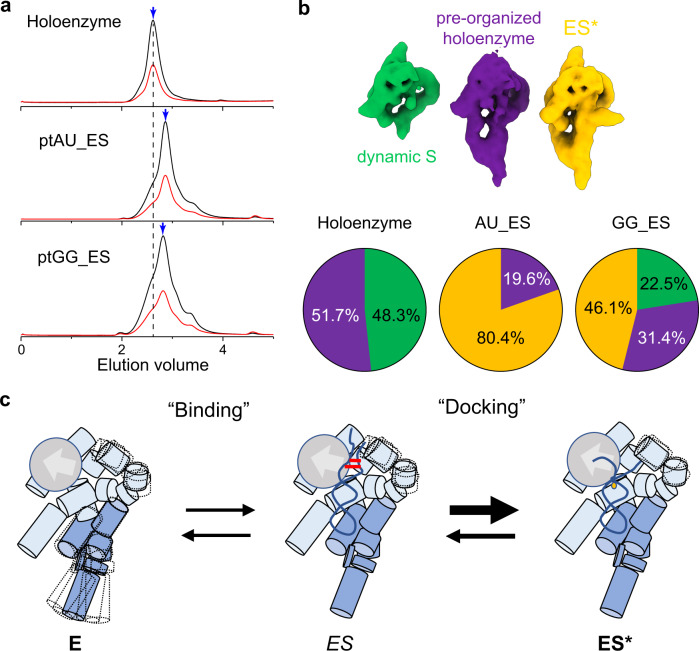
Dynamics of the RNase P holoenzyme and ES complex. **a** Folding of *E. coli* RNase P holoenzyme and assembly with various ptRNA substrates. Size-exclusion chromatograms of RNase P holoenzyme in the presence of Mg^2+^ (top) and AU_ES* and GG_ES* ptRNA-bound complexes assembled in the presence of Ca^2+^. UV absorbance profile at 260 nm (black line) and 280 nm (red line). Peak fraction under the blue arrow was used for cryoEM study. **b** Distribution of conformational states observed for RNase P holoenzyme and ptRNA-bound complexes. **c** Model for molecular recognition of ptRNA by RNase P. A flexible S-domain undergoes a conformational change upon binding ptRNA, which may occur by kinetic trapping or classic induced fit to form an intermediate ES complex. ES undergoes another conformational change to form a stable ES* complex in which the 3′ RCCA of ptRNA base pairs are unwound and the 5′ leader and cleavage site are recognized by the RNase P active site.

We were able to reconstruct two classes of cryoEM maps from the holoenzyme sample (Supplementary Fig. 6a–d). Both classes of holoenzymes were observed with rnpA bound and resolved to 3.1 Å resolution (Supplementary Fig. 6d). One class lacks well-resolved features in the cryoEM map to account for most of the S-domain (G127 to U231). A second class consists of free holoenzyme particles in which the S-domain and C-domain are folded and in approximately the same position observed in the ES* complex. Comparison of both cryoEM maps reveals that the tertiary dinucleotide stack between A118 and A233 could be involved in minimizing the conformational flexibility of the S-domain (Supplementary Fig. 6e, f). Using the two holoenzyme references and the ptRNA-ES* structure, we further sorted each ptRNA-ES* dataset into these three states (Fig. 5). Notably, the distribution of the three classes identifies a significantly larger proportion of ptRNA-bound RNase P for the ptRNA_AU (~80% ES* complex) over ptRNA_GG (~46% ES* complex) dataset. This finding is consistent with the kinetic, binding, and gel shift data (Figs. 2, 3), where ptRNA_GG demonstrates a slower binding step than ptRNA_AU does.

Superpositioning of the *E. coli* holoenzyme and the two ptRNA-ES* structures, as well as the *T. maritima* EP structure, unveiled critical insights into the enzymatic process for RNase P. Local resolution as well as 3D variability analysis from cryoEM particles demonstrated additional dynamic information for the holoenzyme and the ES* complex (Supplementary Fig. 7 and Supplementary Movies 1, 2). The peripheral structural elements, including P1, P2, P15-17 helices and the whole S-domain, in the holoenzyme display a larger degree of conformational heterogeneity as compared to the ES* complexes (Supplementary Fig. 7a and Supplementary Movies 1). Morphing of the holoenzyme and ES* structures provides the premise to propose a model for RNase P processing. In this model, binding of a folded ptRNA on the T-loop would induce motions in the S-domain that would position ptRNA into the active site concertedly with the L15 internal loop capturing the 3′ RCCA (Supplementary Movie 3). Notably, several residues around the active site, such as A248, G332 and A333, all have well defined map features in the holoenzyme structure (Supplementary Fig. 8a), indicating a pre-organized stable pocket for 5′ leader sequence binding. The conformational heterogeneity of the S-domain would help to accommodate different sequences and base pairs in the acceptor stem of ptRNA from diverse substrates. The lifting motion observed from the S-domain, together with the pre-shaped A248, could allow A248 to separate single-base-pairs of the acceptor stem to allow the individual, single-strands of RNA to occupy the 5′-leader binding pocket and the L15 internal loop (Fig. 5b).

RNase P is proposed to use two active site Mg^2+^ ions for catalysis^17,55^, one Mg^2+^ (M_A_) coordinates the universally conserved bulged U (U69 in *E. coli*) in helix P4 in the C-domain and the pro-Rp oxygen of the reactive phosphoryl group. A second Mg^2+^, M_B_, binds in the adjacent J3/4 region and positions the water nucleophile for phosphodiester bond hydrolysis^10,56,57^. We observed a similar inner-sphere ion coordination of an M_A_ ion at the G1 phosphate group in both ES* complexes (Fig. 4a). Like the *T. maritima* EP complex structure the M_B_ ion is not observed. Moreover, we did not observe strong cryoEM map features for the key catalytic residue U69 in our ES* complexes although both biochemical and structural data from the *T. maritima* EP complex show the O4 of the P4 bulged U coordinates the M1 ion (Supplementary Fig. 8b–d). Notably, this U undergoes rotation upon substrate binding facilitating coordination of M1 in yeast RNase P^12^. As discussed below these observations provide insight into functional divalent metal binding and the potential basis for inhibition by Ca^2+^.

## Discussion

The kinetic analyses and structures of ES* complexes with ptRNA^Met^_GG and ptRNA^Met^_AU significantly advance our understanding of how RNase P acts as a multiple substrate endonuclease by resolving inhibitory structure and accommodating sequence variation at its cleavage site. Importantly, ptRNAs with G(−2)G(−1) and other dinucleotides that inhibit formation of ES* are nonetheless highly represented in among *E. coli* RNase P substrates. Of the 86 ptRNA encoded in the *E. coli* genome ~1/3 contain the four least favorable combinations of nucleotides at N(−1)N(−2) (Fig. 2e). RNase P recognition of ptRNA depends on a combination of determinants and anti-determinants, and lengthening of the acceptor stem by N(−1)N(−2) pairing is only one determining factor for *k*_cat_/*K*_m_^58^. Nonetheless, there is evidence that structural anti-determinants play a role in polycistronic ptRNA processing by *E. coli* RNase P. Remarkably, all seven valine tRNAs in *E. coli* require RNase P for separation from their primary polycistronic transcripts, which processes them in a 5′ to 3′ directional manner^59^. In the valVW operon a stem-loop in 5′ leader of tRNA^valV^ inhibits RNase P cleavage resulting in enhanced cleavage of the 3’ most tRNA^valW^ thus enforcing directional processing^24,60^. In the valU operon, the most 5′ ptRNA^valU^ is processed last and has GG at N(−1)N(−2), the internal tRNA^valX^ and tRNA^valY^ have GU, while the 3′ tRNA^lysV^ that is processed first has a near optimal UC dinucleotide. Together with inhibition of processing by 3′ trailer sequences at N(−1)N(−2) are likely to contribute to directional processing of polycistronic ptRNA which is an essential function of *E. coli* RNase P^61^.

Bacterial RNase P is assumed to employ a canonical two metal ion mechanism, however, structures of bacterial and eukaryotic RNase P bound to tRNA product or ptRNA only provide evidence for a single putative active site Mg^2+^ ion (M_A_ in the two metal ion paradigm). Intriguingly, in the *E. coli* ES* complex trapped using Ca^2+^ we also observe density for only M_A_. There is emerging data that dynamic fluctuations in metal binding and occupancy influence phosphoryl transfer enzyme activity^62–64^. It is plausible therefore that the missing M_B_ may be the final trigger for RNase P catalysis, as differences in the affinities of active site metals can control enzyme reactivity^63^. Ca^2+^ has been widely used to trap ES complex where it can replace one or more active site Mg^2+^ ions^65–69^, but can also lead to defects in other enzyme functions particularly for ribozymes. For example, Ca^2+^ does not support Group I intron ribozyme catalysis as it limits a conformational change docking the substrate helix in the active site^70^. Even partial replacement of Mg^2+^ with Ca^2+^ results in stable misfolded species of the *Sc*.ai5γ Group II intron ribozyme^71^. A second class of Ca^2+^ competes with or alters active site Mg^2+^ binding^69^. Thus, *E. coli* RNase P fortuitously avoids misfolding in Ca^2+^ which can also replace M_A_, but unlike Mg^2+^ does not fully engage U69 which may further weaken M_B_ and slow cleavage.

Although the structures of eukaryotic and archaeal RNase P appear to be largely pre-organized for substrate binding, several lines of evidence indicate the bacterial enzyme is more dynamic. Small angle X-ray scattering, SHAPE, and molecular modeling revealed intrinsic conformational flexibility in P RNA consistent with reorganization of the S- and C-domains and undocking of peripheral tertiary interactions^72^. In-cell SHAPE-Seq data for *E. coli* P RNA shows surprisingly high reactivity in a large region predicted to be engaged in structure that encompasses nucleotides 226 to 233, and 129–139. These nucleotides map to the connection between the S-domain and C-domain^73^. The reactive positions include A233 which interacts with ptRNA in the T-loop and may communicate binding to the C-domains via inter-domain motion. L9 and A118 are also highly reactive consistent with the dynamic behavior we document involving P9 occurring in vivo. Time resolved fluorescence resonance energy transfer between labeled rnpA protein and ptRNA 5′ leader showed unusually extended RNA conformation with reduced dynamics at nucleotides proximal to the cleavage site^49^. The authors proposed RNase P acts as a wedge to separate the 5′ from the 3′ terminus of the ptRNA and to position the cleavage site in the catalytic core consistent with the cryoEM and biochemical data presented here. Interestingly, early studies by Pomeranz Krummel et al. using synthetic substrates with inter-strand crosslinks near the cleavage site indicated that restricting helical dynamics negative impacts efficient processing by *E. coli* RNase P^74^. In sum, the cryoEM results are consistent with a dynamic ensemble of free RNase P structures with motion between the domains and significant dynamics involving the S-domain. Binding of ptRNA redistributes the ensemble of RNase P enzymes, and intermolecular and intramolecular interactions including binding of active site metal ions drives the formation of ES*.

The energy cost of redistributing the multiple states adopted by the free enzyme into the active conformation is likely paid for by formation of favorable intramolecular RNA-RNA contacts most likely involving L9-P1 and P15/16, as well as intermolecular interactions with ptRNA. Presumably the energetic cost for stabilizing RNase P is similar for all substrates, and so will not necessarily provide specificity between alternative ptRNAs^75^. However, the linkage of ES* formation to recognition of specific elements of ptRNA structure would act to distinguish between cognate and non-cognate RNAs. In the ES* complex the bound ptRNA is in a distorted conformation that cannot easily dock directly into a static RNase P particularly for substrates requiring extended acceptor stem unwinding. The inability of ptRNAs to adopt conformations matching the active site of RNase P necessitates conformational changes that present additional free energy barriers to formation of ES*. Since these barriers can be different for different ptRNAs, the proposed induced fit mechanism could contribute to specificity (i.e., the relative *k*_cat_/*K*_m_ for alternative substrates). Mutually induced conformational changes are observed in protein-protein and protein-ligand interactions, DNA-protein interactions and RNA-protein interactions^76^, and ribonucleoprotein assembly, where the involvement of RNA dynamics is widespread^77^. Relevant examples involving tRNA include induced fit mechanisms that contribute to tRNA selection by the ribosome^78^, aminoacyl tRNA synthetase enzymes^79^, and tRNA modifying enzymes that partially unwind the tRNA anticodon stem^80^.

An important consequence of coupling of folding and binding with unwinding of ptRNA is that knowledge of the free energy cost or contribution of these processes is needed to account for the energetics of binding. This information is essential to interpret differences in the rates of processing between alternative substrates, and the effects of mutations in P RNA and rnpA. The interplay between enzyme flexibility and substrate recognition is important for the function of a multitude of enzymes. Understanding these linkages remains a significant obstacle both to our understanding of specificity and reliable prediction of cognate substrates and processing rates in vivo. RNase P binding and specificity encompass questions that are foundational for understanding enzymology in general. These results provide a biochemical and structural context for understanding the multiple steps that lead up to the RNase P-catalyzed ptRNA cleavage reaction that contribute to alternative substrate selection, site specificity, and high catalytic efficiency.

## Methods

### Preparation of substrate RNAs and RNase P

The *E. coli* C5 protein was overexpressed, affinity purified by the NEB IMPACT system as a chitin-binding domain fusion with subsequent removal of the tag by intein cleavage, dialysis and concentration^81^.

The DNA templates for synthesis of RNA molecules by in vitro transcription were either prepared by PCR amplification or prepared as plasmids transformed into and maintained in competent *E. coli* cells. The randomized substrate pool for HTS-Kin experiments, referred to as ptRNA^met^(N(−6) to N(−1))_21C in the text, is based on non-initiator ptRNA^Met608^ and was prepared by using DNA primers incorporating randomized mutations at N(−6 to −1) region for PCR amplification of the cloned DNA template^37^. The DNA templates of mutant ptRNA substrates were prepared by using DNA primers incorporating desired leader sequence and modifications of 5′ and 3′ end for PCR amplification.

The following PCR primers were used (5′ leader sequence is underlined):

Forward primers:

21C randomization oligos: TAA TAC GAC TCA CTA TAG CAT GCA CGA AAG TGC GTG CTA TGA ANN NNN NGG CTA CGT AGC TCA GTT GG

AU substrate: TAA TAC GAC TCA CTA TAG AAT TCT ATG GCT ACG TAG CTC AGT TGG

GG substrate: TAA TAC GAC TCA CTA TAG AAT TCT GGG GCT ACG TAG CTC AGT TGG

AU_T5 substrate: TAA TAC GAC TCA CTA TTA ATG GCT ACG TAG CTC AGT TGG

Reverse primers:

WT: TGG TGG CTA CGA CGG GAT TC

D3 mutant: GGC TAC GAC GGG ATT CGA AC

The sequence of P RNA was cloned into pUC18 vector and amplified by NEB 5-alpha competent *E. coli* cells. Cells were harvested and plasmids were extracted with ZymoPURE II Plasmid Midiprep Kit (Zymo Research). The plasmids were linearized with BbsI restriction enzyme (NEB) to yield the template for in vitro transcription. The P RNA and ptRNAs were synthesized by in vitro transcription using T7 RNA polymerase purified by overexpression, and the DNA templates described above in reactions in a volume of 400 μL containing 40 mM Tris-HCl pH 8, 16 mM MgCl_2_, 2 mM spermidine and 10 mM dithiothreitol (DTT)^82^. The transcribed products were purified by 6% denaturing polyacrylamide gel electrophoresis. RNA molecules were passively eluted from gel slices to elution buffer (10 mM Tris-HCl pH8, 100 mM NaCl, 1 mM EDTA, 0.1% SDS) and recovered by phenol/chloroform extraction followed by ethanol precipitation.

### High-throughput sequencing kinetics (HTS-Kin)

HTS-Kin simultaneously measures the relative *k*_cat_/*K*_m_ values (k_rel_) for thousands of ptRNA substrates in a single in vitro RNase P reaction. Briefly, the ptRNA^met^(N(−6) to N(−1))_21C randomized substrate population was reacted in vitro with *E. coli* RNase P. The unreacted ptRNA was purified from specific time points, and the time-dependent change in the distribution of individual ptRNA sequence variants was determined by Illumina sequencing. Internal competition kinetics were used to calculate *k*_rel_ and the genomically encoded ptRNA^Met82^ 5′ leader sequence was used as a reference.

The determination of relative rate constants for *E. coli* RNase P processing of ptRNA^Met^ by HTS-Kin^37,40^. Briefly, multiple turnover reactions using the ptRNA^met^(N(−6) to N(−1))_21C randomized substrate population were performed as described below, but scaled up tenfold to provide sufficient material for preparation of samples for RT-PCR and Illumina sequencing. Aliquots were taken during the time course at ~5–30% conversion of the input ptRNA, and the residual precursor population resolved from products by 15% denaturing PAGE. The remaining substrates were excised, eluted, and purified as described above. Complementary DNA libraries for Illumina sequencing were prepared from the unreacted ptRNA that was recovered from PAGE purification. The methods for first strand cDNA synthesis, PCR, and preparation of Illumina sequencing samples were identical to previous experiments using ptRNA^met^ randomized pools^19^ and sequenced in a single lane.

All reads were aligned and sorted according to their index tag. The relative rate constant for individual sequence variants (*S*_*i*_) were calculated from these data using the following equation.

1 $${k}_{rel}\,=\,{{\mathrm{ln}}}\,\frac{(1\,-\,{{{\rm{f}}}})}{\frac{{{{{\rm{R}}}}}_{{{{\rm{i}}}},0}}{{{{{\rm{R}}}}}_{{{{\rm{i}}}}}}\left({\sum }_{1}^{{{{\rm{i}}}}}\frac{{{{\rm{R}}}}}{{{{{\rm{R}}}}}_{0}}\right)}\bigg/{{\mathrm{ln}}}\,\frac{(1\,-\,{{{\rm{f}}}})}{{\sum }_{1}^{{{{\rm{i}}}}}\frac{{{{\rm{R}}}}}{{{{{\rm{R}}}}}_{0}}}$$

where *R*_i_ is the ratio S_2_/S_1_ determined at remaining total substrate *f* and *R*_i,0_ is the ratio S_2_/S_1_ at the start of the reaction. These ratios are calculated from the number of Illumina sequence reads at the start of the reaction and at specific fractions of total substrate reacted.

### Sequence specificity modeling

The sequence specificity of *k*_rel_ cannot be adequately described by a simple position weight matrix (PWM) model (Supplementary Fig. 2c)^19,37^. The limitation is that they assume bases in the binding sequence contribute in an independent and identically distributed, non-interacting manner. However, contributions of individual nucleotides at a particular position in the binding site are typically dependent on their local sequence context. Accordingly, we use a model of sequence specificity that adds an interaction term to the independent variables that describes the interaction between bases (Pairwise Interaction Matrix, PIM). A basic assumption of these new terms is that they are independent and identically distributed, nonetheless they permit a simple quantitative assessment of coupling between the contributions of individual positions to RNase P binding and cleavage. Briefly, the values for PIM are based on the following coefficients: a_*i*_, c_*i*_, g_*i*_, u_*i*_, (−6 ≤ *i* ≤ −1). Interaction coefficients (*I*_n_) included in the model results in significantly better fits to the experimental data^19,37^.

2 $${{{\rm{ln}}}}\left({k}_{{rel}}\right) \sim \mathop{\sum }\limits_{i\,=\,3}^{8}\left({a}_{i}{{{{\rm{A}}}}}_{{{{\rm{i}}}}}\,+\,{c}_{i}{{{{\rm{C}}}}}_{{{{\rm{i}}}}}\,+\,{g}_{i}{{{{\rm{G}}}}}_{{{{\rm{i}}}}}\,+\,{u}_{i}{{{{\rm{U}}}}}_{{{{\rm{i}}}}}\right)\,+\,\mathop{\sum }\limits_{i\,=\,1}^{n}{\alpha }_{n}{{{{\rm{I}}}}}_{{{{\rm{n}}}}}$$

Interaction terms with T values larger than 3.5 were selected in each round of regression, and those that reflect the null hypothesis rejected.

### Analysis of multiple turnover kinetics

The 5′ end phosphates of ptRNA substrates were removed by Quick CIP (NEB) prior to labeled ^32^P. The treated ptRNAs were 5′ end radio-labeled with γ-^32^P-ATP (PerkinElmer) by T4 polynucleotide kinase (NEB). The excess of free nucleotides was removed by running the reaction in a 6% denaturing PAGE and the radio-labeled ^32^P-ptRNAs were eluted from the gel slices and recovered by ethanol precipitation, as described above.

For multiple turnover reactions, (a) 20 nM–2 μM non-labeled “cold” ptRNA substrates with ~1 nM of radio-labeled ptRNAs and (b) 2 nM P RNA were unfolded and refolded separately in 50 mM Tris-HCl pH8, 100 mM NaCl and 0.005% reduced Triton X-100 at 95 °C for 3 min followed by 37 °C for 10 min on a thermal cycler (MJ research PTC-200). MgCl_2_ was added to each tube with a final concentration of 17.5 mM after RNA refolding and the solutions were incubated at 37 °C for another 10 min. To make sure all the P RNA were bound, 10 nM of C5 protein was then added to the P RNA for assembly of the RNase P holoenzyme, and the tubes were incubated at 37 °C for an additional 10 min. Substrate cleavage reactions were initiated by adding an equal volume of solutions containing substrate and enzyme to achieve the final concentration of 1 nM enzyme and 10 nM–1 μM substrate. Aliquots were taken at selected time points and quenched immediately with equal volume of formamide loading dye incorporating 100 mM EDTA. Remaining substrates and products were resolved by a 15% PAGE, after pre-running for 30 min at 80 W. The gel was dried for 2 h in a gel drier (Model 583, Bio-Rad) and exposed to a phosphor screen (GE Healthcare) overnight. The screen was scanned by Amersham Typhoon RGB phosphor-imager (GE Healthcare) and the readings of substrate and product bands were quantified by ImageQuant TL 8.2 (GE Healthcare). For substrates showing mis-cleavage the intensity of all product bands was summed to calculate the fraction of substrate reacted.

Origin 8.5 (OriginLab) was used for the data fitting. The reacted fraction was calculated as the volume of product band divided by the sum of volumes of substrate and product bands. The reacted fraction between 0 and 0.1 was plotted against time (in second) and fit to a linear regression equation. The observed initial reaction rate, *v*_*initial*_ for this reaction can be approximated as the slope of the fitting line multiplying the initial substrate concentration, [S]. The concentration of radio-labeled substrate was negligible, and the final concentrations of cold substrate were plugged in as [S]. At each [S], *v*_*initial*_ were measured at least three times. The averages of *v*_*initial*_ values at different [S] were plotted against [S] and the plot was fit to the Michaelis-Menten Equation with a weighting method based on the standard deviation of the multiple replicates

3 $${v}_{{initial}}\,=\,{{{{\rm{k}}}}}_{{{{\rm{cat}}}}}{[{{{\rm{E}}}}]}_{0}\frac{[{{{\rm{S}}}}]}{{{{{\rm{K}}}}}_{{{{\rm{m}}}}}\,+\,[{{{\rm{S}}}}]}$$

where ${{{{\rm{k}}}}}_{{{{\rm{cat}}}}}$ is the catalytic rate constant, ${[{{{\rm{E}}}}]}_{0}$ is the initial enzyme concentration and ${{{{\rm{K}}}}}_{{{{\rm{m}}}}}$ is the Michaelis constant. Therefore, ${{{{\rm{k}}}}}_{{{{\rm{cat}}}}}$ and ${{{{\rm{k}}}}}_{{{{\rm{cat}}}}}/{{{{\rm{K}}}}}_{{{{\rm{m}}}}}$ were derived. The experimental error for ${{{{\rm{k}}}}}_{{{{\rm{cat}}}}}$ and ${{{{\rm{k}}}}}_{{{{\rm{cat}}}}}/{{{{\rm{K}}}}}_{{{{\rm{m}}}}}$ values listed in Table 1 were generated from the standard errors of the fitting.

### Gel mobility shift analysis of RNase P-ptRNA binding affinity and complex stability

The dissociation constant, K_d_, for ptRNA_AU and ptRNA_GG binding to *E. coli* RNase P was determined by electrophoretic mobility shift analysis (EMSA). 2.5–200 nM of P RNA and ~1 nM of ^32^P-labeled ptRNA were refolded separately in 90 mM Tris-HCl pH8, 100 mM NaCl, and 7% glycerol, 0.005% reduced Triton X-100 and small amount of xylene cyanol by incubating at 95 °C for 3 min followed by 37 °C for 10 min. CaCl_2_ was added to a final concentration of 3 mM, and samples were incubated at 37 °C for another 10 min. rnpA protein was then added to the pre-folded P RNA to a final concentration of 1.5-fold of the P RNA concentration. After an additional incubation for 10 min at 37 °C, equivalent volumes of enzyme and substrate were mixed to initiate binding. Mixtures were incubated at 37 °C for 3 min and on ice for 15 min. ES and ES* complexes were separated from free substrates by a 7% native polyacrylamide gel that incorporates of 90 mM Tris-HCl pH8, 100 mM NaCl, 3 mM CaCl_2_. The gel was pre-run for 30 min at 60 W and then samples were loaded into the gel. It was run at 60 watts for 1.5 h, with the gel tank sitting in ice-water bath to keep the temperature of the gel lower than 25 °C. The gel was dried and exposed to a phosphor screen, which was then scanned in a similar manner as described above. The ES+ES* and free substrate bands were quantified with ImageQuant TL 8.2. The bound percentage of total substrate population (*y*) was calculated as volume of ES+ES* band divided by the sum of volume of ES+ES* and free substrate bands and plotted against enzyme concentration, [E]. Data was fit to

4 $$y\,=\,{{{{\rm{B}}}}}_{{{{\rm{max}}}}}\,*\, \frac{\left[{{{\rm{E}}}}\right]}{{{{{\rm{K}}}}}_{{{{\rm{d}}}}}\,+\,\left[{{{\rm{E}}}}\right]}$$

where *B*_*max*_ is the maximum observed fraction bound. At least three repeats for each substrate were done and the average and standard deviation of them were reported in Table 1.

Based on previous experiment, the stability and fraction of ES and ES* complexes were further optimized. ES and ES* complexes with 100 nM of RNase P were prepared as described above, and an aliquot of the solution was loaded into a 7% native polyacrylamide gel and noted as t = 0. Gel was set to start running before unlabeled substrate was added in the ES + ES* mixture with a final concentration of 500 nM. Aliquots were taken at selected time points and loaded immediately into the running gel, which was subsequently dried and exposed to phosphor screen. The screen was scanned and signals of bound and free ptRNA were quantified as described above.

### Size-exclusion chromatography and CryoEM sample preparation

Purified *E.coli* P RNA and pre-tRNA substrate (AU and GG) were refolded in 50 mM Tris pH 8.0, 100 mM NaCl buffer in a PCR thermal cycler for 3 min at 95 °C followed by 10 min at 37 °C. After refolding, 10 mM of CaCl_2_ or MgCl_2_ was added to the RNA samples and incubated at 37 °C for 10 min. For RNase P holoenzyme, five-fold excessive concentration of purified P *E.coli* P protein was then added to the P RNA sample and incubated at 37 °C for 10 min. The RNase P holoenzyme complex was subsequently polished using size-exclusion chromatography (SEC) using SRT-10 SEC-300 column (Sepax Technologies) in 50 mM Tris pH 8.0, 100 mM NaCl, 10 mM MgCl_2_ running buffer. For ES* complexes, equivalent concentrations of holoenzyme and pre-tRNA substrate were mixed and loaded onto an SRT-10 SEC-300 column (Sepax Technologies) using 50 mM Tris pH 8.0, 100 mM NaCl, 10 mM CaCl_2_ as the running buffer.

### CryoEM data collection and image processing

Peak fractions of SEC corresponding to ES* complexes were used to prepare cryoEM grids. Quantifoil grids (R1.2/1.3 300 mesh) were glow-discharged and coated with graphene oxide thin layer flakes following the protocol (figshare. Media. 10.6084/m9.figshare.3178669.v1)^83^. The cryoEM specimens were prepared using an FEI Vitrobot Mark IV with 3.5 μl of SEC peak fraction. Grids were blotted for 3 s with blot force −5 in 100% humidity at 4 °C prior to plunge freezing. The frozen-dehydrated grids were transferred to a Titan Krios (Thermo Fisher Scientific) transmission electron microscope equipped with a Gatan K3 direct-electron counting camera and BioQuantum energy filter for data acquisition. Movies of the specimen were recorded with a nominal defocus setting in the range of −1.0 to −3.0 μm using Leginon with beam-tilt image-shift data collection strategy^84^. The movie stacks were collected in super-resolution mode of the K3 camera at the magnification yielding a physical pixel size of 1.058 Å/pixel. Each stack was exposed for 3 s, with each frame exposed for 0.05 s, resulting in a 60-frame movie. The total accumulated dose on the specimen was 52.93 e/Å^2^ for each stack.

For RNase P holoenzyme, movies of the specimen were recorded with a nominal defocus setting in the range of −1.0 to −2.5 μm using Latitude with beam-tilt image-shift data collection strategy with a 2 × 2 pattern. The movie stacks were collected in super-resolution mode of the K3 camera at a nominal magnification of 105,000 yielding a physical pixel size of 0.872 Å/pixel. Each stack was exposed for 3.09 s, with each frame exposed for 1/13 s, resulting in a 40-frame movie. The total accumulated dose on the specimen was 53.3 e/Å^2^ for each stack.

Each movie stack was processed on-the-fly using CryoSPARC live (version 3.0.0)^85,86^. The movie stacks were aligned using patch motion correction with a F-crop factor of 0.5. The contrast-transfer function (CTF) parameters of each particle were estimated using patch CTF. Particles were autopicked using a 150 Å gaussian blob. The numbers of bin2 particles selected after 2D classification are included in Supplementary Table 1. The initial 3D volume and decoys were generated using ab initio reconstruction. An even set of superclasses from rebalancing good 2D classes with a minibatch size of 1000 was used to generate three classes of initial 3D volumes. And junk 2D classes were used to generate three 3D decoys. The particles after 2D clean up were submitted to one round of heterogeneous refinement with these ab initio 3D volumes and decoys. Based on the coordinates and angular information of these particles, bin1 particles of the 3D class with well-resolved 3D features were re-extracted from the dose-weighted micrographs. The final particle set was subjected to non-uniform 3D refinements, followed by local 3D refinements, yielding final maps with reported global resolutions using the 0.143 criterion of the gold-standard Fourier shell correlation (FSC) (Supplementary Table 1). The half maps were used to determine the local resolution of each map and focused classification was performed using Relion 3.0 and 3D classification (beta) in CryoSPARC 3.3^86–88^.

### Model building and refinement

The initial models *E.coli* P RNA and pre-tRNA substrate were manually assembled and refined in Coot^89^ using the crystal structure of *T. maritima* RNase P complex (PDB entry: 3Q1Q)^10^ as the scaffold to connect individual structural components for *E.coli* sequences built using MC-sym pipeline^90^. The initial model for *E.coli* P protein was generated using AlphaFold II^91^. After assembling individual subunits into a single PDB file, the models were refined into the composite map using phenix.real_space_refine in Phenix^92^. Base pair restraints were applied to the RNA components for regions with local resolution less than 4 Å. All statistics for structural models are reported in Supplementary Table 1. Figure panels depicting cryoEM maps or atomic models generated using ChimeraX^93^. Maps colored by local resolution were generated using RELION 3.1^87^.

### Reporting summary

Further information on research design is available in the Nature Research Reporting Summary linked to this article.

## Supplementary information

Supplementary Information

Description of Additional Supplementary Files

Supplementary Movie 1

Supplementary Movie 2

Supplementary Movie 3

Reporting Summary

## Source data

Source Data

## Data Availability

The cryoEM maps of RNase P bound with AU_ptRNA and GG_ptRNA have been deposited into the Electron Microscopy Data Bank under accession codes EMD-26637 and EMD-26636, respectively. The corresponding atomic models have been deposited into Protein Data Bank under accession codes 7UO1 and 7UO0 for AU_ptRNA and GG_ptRNA bound RNase P complexes, respectively. The cryoEM maps of RNase P without ptRNA bound have been deposited into the Electron Microscopy Data Bank under accession code EMD-26640 and EMD-26638, and the corresponding atomic models have been deposited into Protein Data Bank under accession codes 7UO5 and 7UO2, respectively. Source data are provided with this paper.
