# Supplementary material for: Structural and mechanistic basis for recognition of alternative tRNA precursor substrates by bacterial ribonuclease P
Source: Nat Commun. 2022 Aug 31;13:5120. doi: 10.1038/s41467-022-32843-7 (PMC9433436; doi:10.1038/s41467-022-32843-7)

SUPPLEMENTARY INFORMATION

Structural and mechanistic basis for recognition of alternative tRNA precursor substrates by bacterial ribonuclease P

Jiaqiang Zhu[1], Wei Huang[2], Jing Zhao[1], Loc Huynh[1], Derek J Taylor[2,3], Michael E Harris[1]

[1]Department of Chemistry, University of Florida, Gainesville, FL 32608

[2]Department of Pharmacology,

[3]Department of Biochemistry, Case Western Reserve University School of Medicine, Cleveland, OH 44118

Contents

Supplementary Fig. 1 | 21C pool kinetics and comparison of technical replicates. 2

Supplementary Fig. 2 | Distribution of k_{rel} values for ptRNAs with different nucleotides at N(-2)N(-1) and sequence specificity modelling. 3

Supplementary Fig. 3 | Comparison of the effect of mutation at N(-4) and N(-2) on k_{rel} 4

Supplementary Fig. 4 | Kinetics and binding affinity of ptRNA_AU and ptRNA_GG. 6

Supplementary Fig. 5 | CryoEM structural analysis of AU_ES* and GG_ES* complexes 7

Supplementary Fig. 6 | CryoEM maps and structures of RNase P holoenzyme 8

Supplementary Fig. 7 | Local resolution analysis for RNase P holoenzyme, AU_ES* and GG_ES* complexes. 9

Supplementary Fig. 8 | Comparisons of structure models across holoenzyme, ES* and EP states for bacterial RNase P complexes. 10

Supplementary Table 1 | CryoEM data collection, processing and model refinement statistics for *E. coli* RNase P holoenzyme and ES* complexes 12

Supplementary Figure 1a uncropped 13

Supplementary Figure 4c uncropped 14

Supplementary Fig. 1 | 21C pool kinetics and comparison of technical replicates.

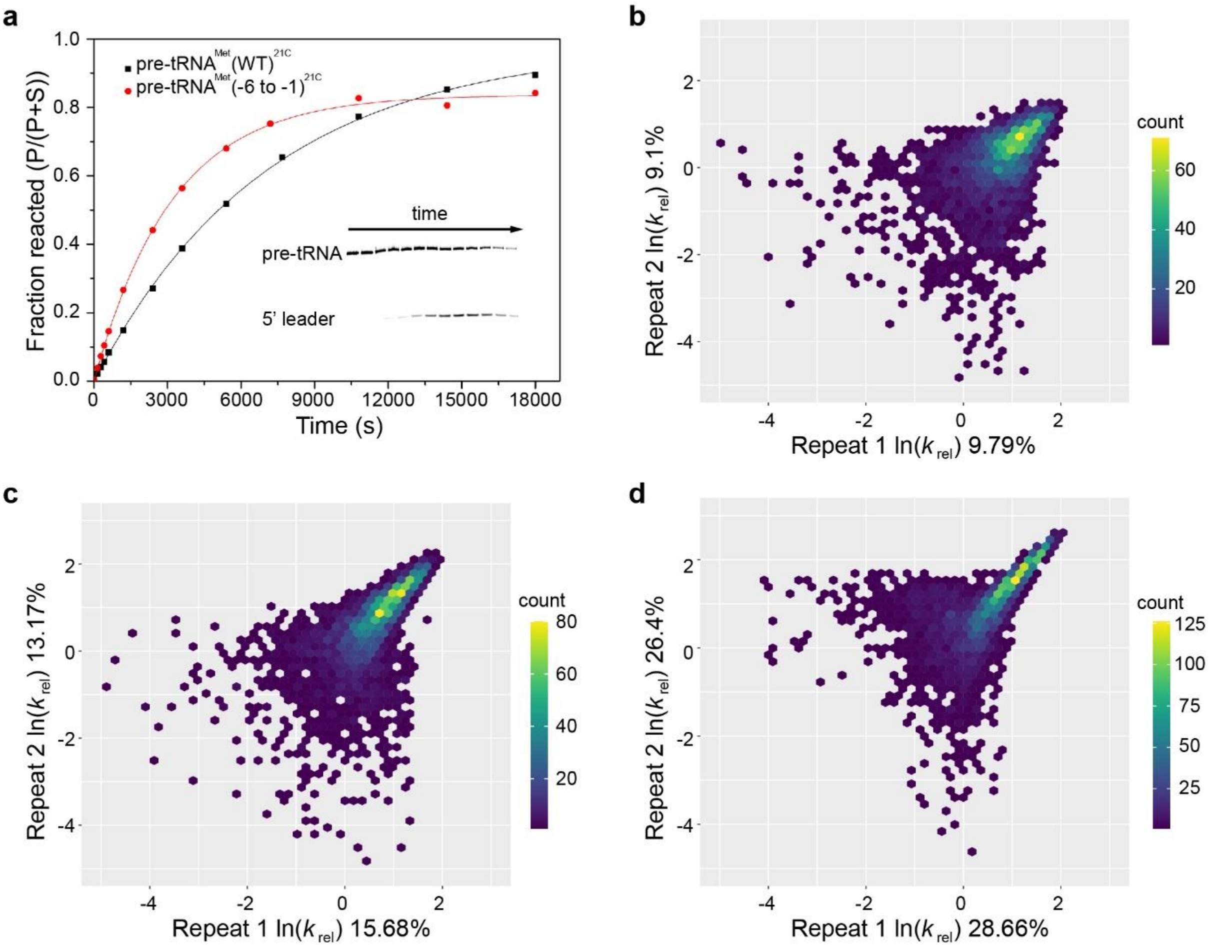

a, Time course for cleavage of ptRNA_21C (black) and the ptRNA_21C pool in which nucleotides N(-1) to N(-6) are randomized (red). The v_{obs} at 1 uM ptRNA^{met}(WT)^{21C} , which has the extra 5' stemloop, is 0.03 ± 0.02 s^{-1} (n=3 independent experiments). This value is comparable to 0.11 ± 0.01 s^{-1} and 0.06 ± 0.01 s^{-1} previously documented for the parent ptRNA^{met82} substrate that lacks the 5' stemloop (Yandek et al. (2013) J Biol Chem. 288(12):8342-8354). Thus, the presence of the 5' stemloop required for HTS-Kin has only a small effect on multiple turnover cleavage by *E. coli* RNase P. The inset shows the resolution of the ptRNA and 5' leader by PAGE using the ptRNA_21C randomized pool that is 5'-^{32}P labelled. **b-d**, Comparison of HTS-Kin results from two independent experiments at similar *F* (percent conversion) illustrating the reproducibility of the rate constant distribution. Excellent correlation between the two data sets is observed with the greatest error observed for the slowest sequence variants. This effect arises due to the small change in sequence reads in the residual substrate population for these species. Source data are provided as a Source Data file.

Supplementary Fig. 2 | Distribution of k_{rel} values for ptRNAs with different nucleotides at N(-2)N(-1) and sequence specificity modelling.

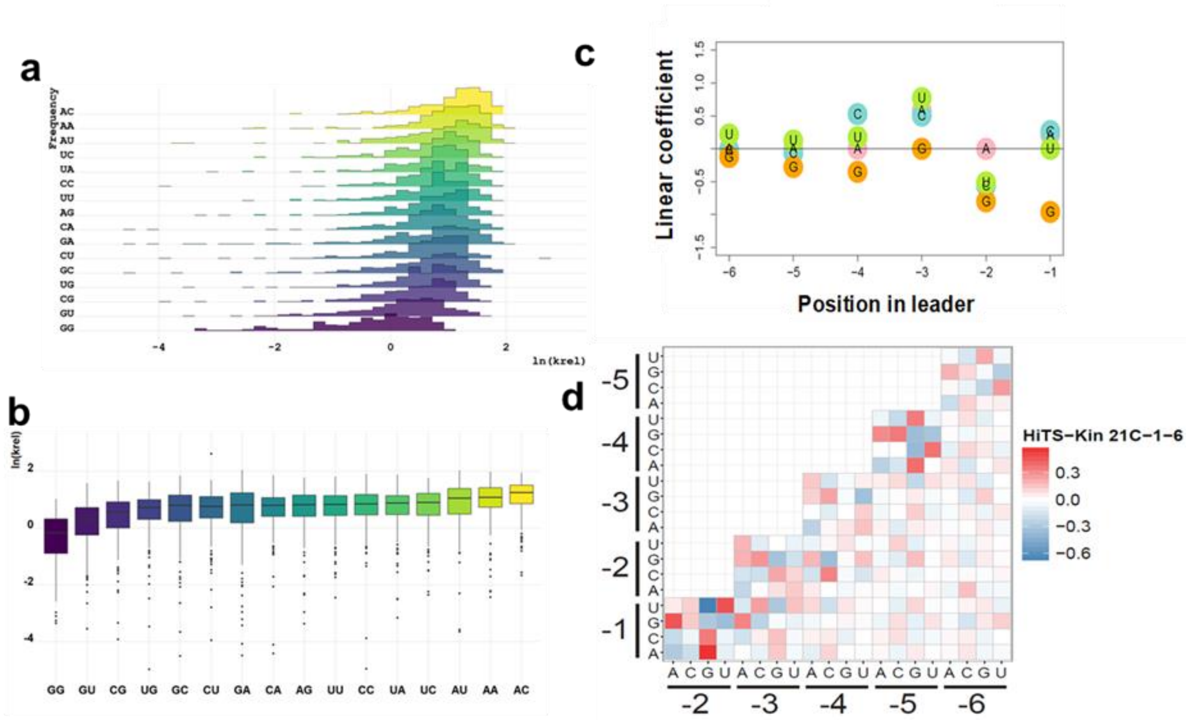

a, Comparison of the distributions of k_{rel} values for ptRNA sequence variants with all possible nucleotides from N(-3) to N(-6) and one of the sixteen possible dinucleotide sequences at N(-2)N(-1). The distributions are overlaid and plotted as the natural log. The columns are shaded according to their relative k_{rel} values. **b**, The distributions shown in panel **a** shown as bar and whisker plots. The center line indicates the mean, the box includes the 25th to 75th percentiles of dataset, whiskers mark the 5th and 95th percentiles, and outliers shown as dots. **c**, PWM values derived from fitting the high-throughput biochemical data as described in Methods. Scoring is relative to the genomically encoded leader (AAAGAU), which contributes a linear coefficient of 0; positive and negative values represent contributions relative to the reference sequences. **d**, PIM values derived from fitting. The identity and position of the nucleotide is indicated on each axis and the absolute value of the difference of the predicted IC values is indicated by the color at the vertex. Source data are provided as a Source Data file.

Supplementary Fig. 3 | Comparison of the effect of mutation at N(-4) and N(-2) on k_{rel}

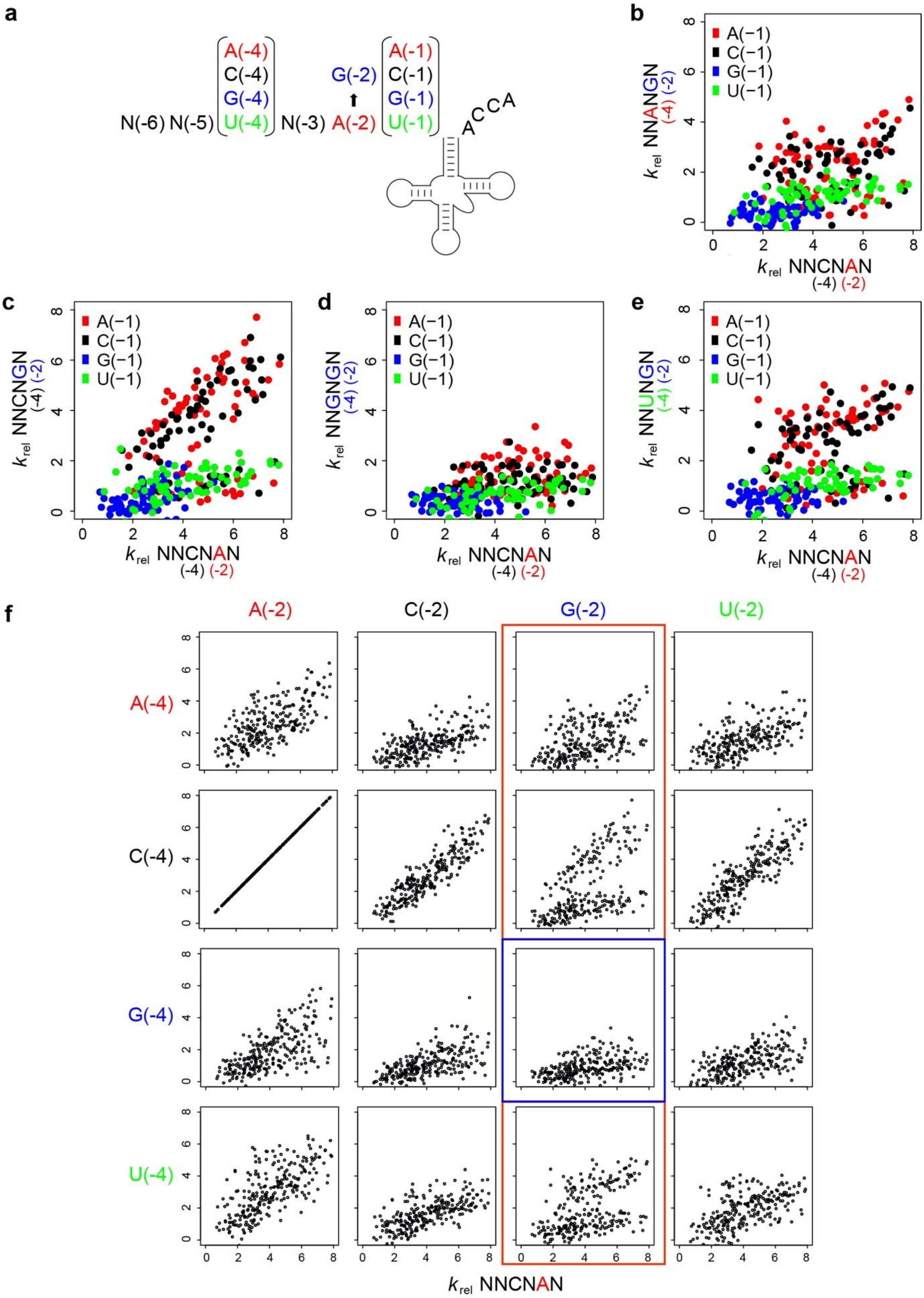

a, Schematic depiction of 21C ptRNA substrate used for HTS-Kin analysis. Nucleotides randomized in the leader sequence pool are numbered. The N(-1), N(-2) and N(-3) positions that determine the magnitude of k_{rel} are highlighted and the colors matching the dot plot comparisons of subsets of the k_{rel} distribution shown in panes. b-f. **b-c**, Dot plot comparison of the effect on k_{rel} of changing A(-2) to G(-2) in the context of different nucleotides at N(-4). The individual points are coloured according to the identity of the nucleotide at N(-1). **f**, Dot plot comparison of all possible combinations of nucleotides at N(-2) and N(-4). The plots highlighted in panels **b-e** are indicated: red box (panels b,c,e) and blue box (panel d). Source data are provided as a Source Data file.

Supplementary Fig. 4 | Kinetics and binding affinity of ptRNA_AU and ptRNA_GG.

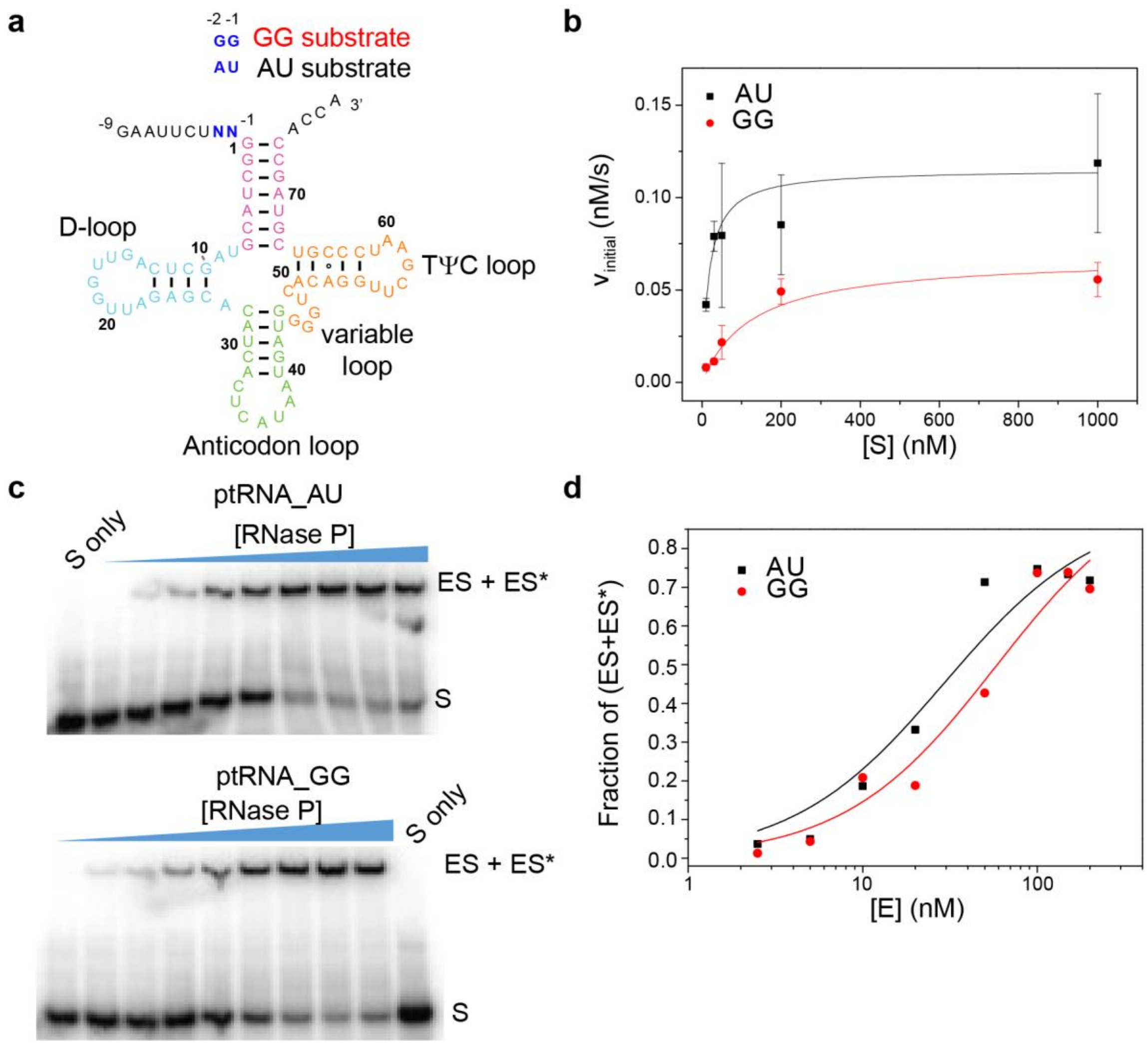

a, Sequence and secondary structure of ptRNA_AU and ptRNA_GG. **b**, Multiple turnover reaction kinetics of ptRNA_AU and ptRNA_GG at limiting substrate concentration showing the difference in k_{cat}/K_m. Data are presented as mean values +/- SEM. (10 nM and 30 nM, n=3 independent experiments; 50 nM, n=8 independent experiments, 200 nM and 1000 nM, n=5 independent experiments) **c**, EMSA analysis of equilibrium binding of ptRNA_AU (top) and ptRNA_GG (bottom). The reactions were performed using 5' ^{32}P-labeled ptRNA incubated with increasing concentrations (2.5-200 nM) of RNase P in 3 mM Ca^{2+} at pH=8. The position of free ptRNA and bound complex are indicated as a mixture of ES and ES*. Three independent experiments were used to determine the mean +/- SEM for binding affinity. **d**, Plot of fraction of ptRNA bound versus concentration of RNase P from EMSA data illustrating similar K_d values (~2-fold difference) for binding of the two substrates (as labelled) to RNase P. Source data are provided as a Source Data file.

Supplementary Fig. 5 | CryoEM structural analysis of AU_ES* and GG_ES* complexes

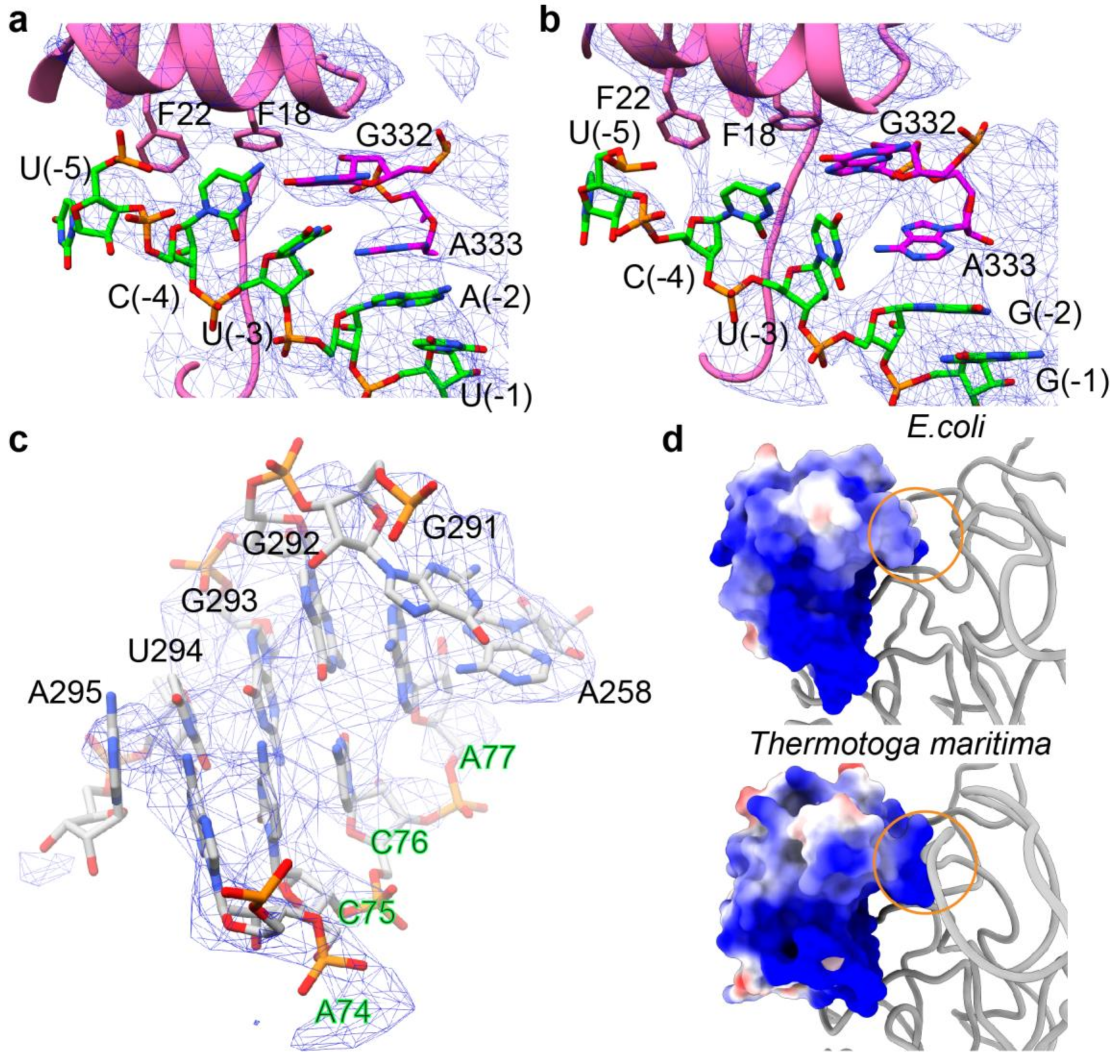

a, CryoEM density for the 5’ leader sequence observed in AU_ES* complex. **b,** CryoEM density for the 5’ leader sequence observed in GG_ES* complex. **c,** Structural model for the 3’ RCCA interactions with L15 internal loop in cryoEM density. **d,** Electrostatic surface rendering of *E.coli* and *T. maritima* rnpA. Surface accounted for R12, R14 and R15 in *T. maritima* rnpA and their counterpart in *E.coli* rnpA is highlighted by an orange circle.

Supplementary Fig. 6 | CryoEM maps and structures of RNase P holoenzyme

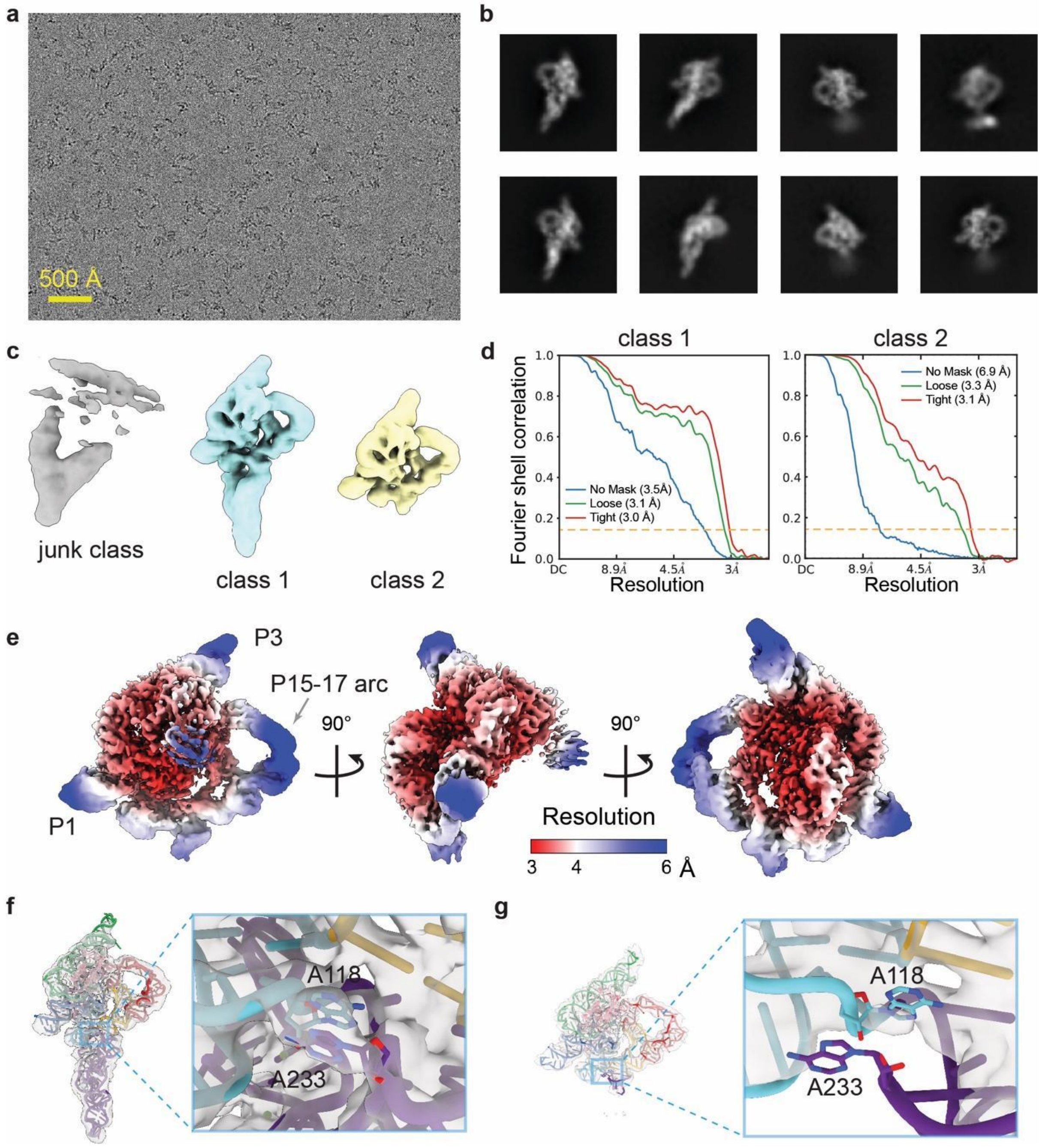

a, Representative cryoEM micrograph of RNase P holoenzyme. The total number of micrographs is documented in Supplementary Table 1. **b,** Representative 2D class averages of RNase P holoenzyme sample. **c,** Ab-initio reconstruction with *k*=3 yields two classes of reference volumes with high-resolution features (individually shown in yellow and cyan) and a junk class (grey). **d,** Gold standard FSC-curve for class 1 and 2 of RNase P holoenzyme cryoEM maps after the final local refinement step. **e,** Local resolution map for the class 2 RNase P holoenzyme particles. **f-g,** Close-up view of cryoEM density around the A118 and A233 dinucleotide stack in both classes of RNase P holoenzyme.

Supplementary Fig. 7 | Local resolution analysis for RNase P holoenzyme, AU_ES* and GG_ES* complexes.

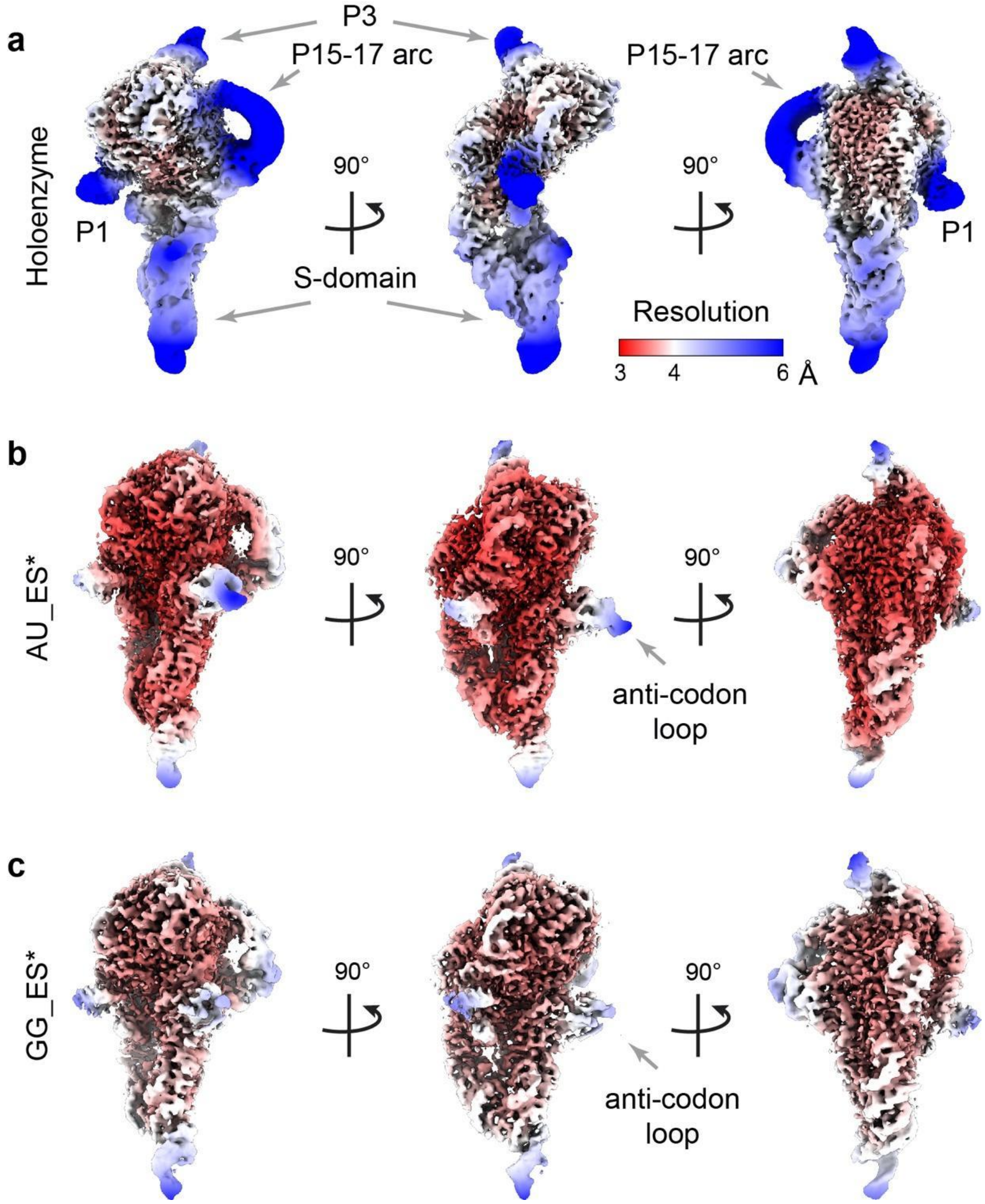

Three views of the cryoEM map for RNase P holoenzyme (**a**), AU_ES* complex (**b**), and GG_ES* complex (**c**). All maps are coloured using local resolution values, correlating to the scale shown in panel **a**.

Supplementary Fig. 8 | Comparisons of structure models across holoenzyme, ES* and EP states for bacterial RNase P complexes.

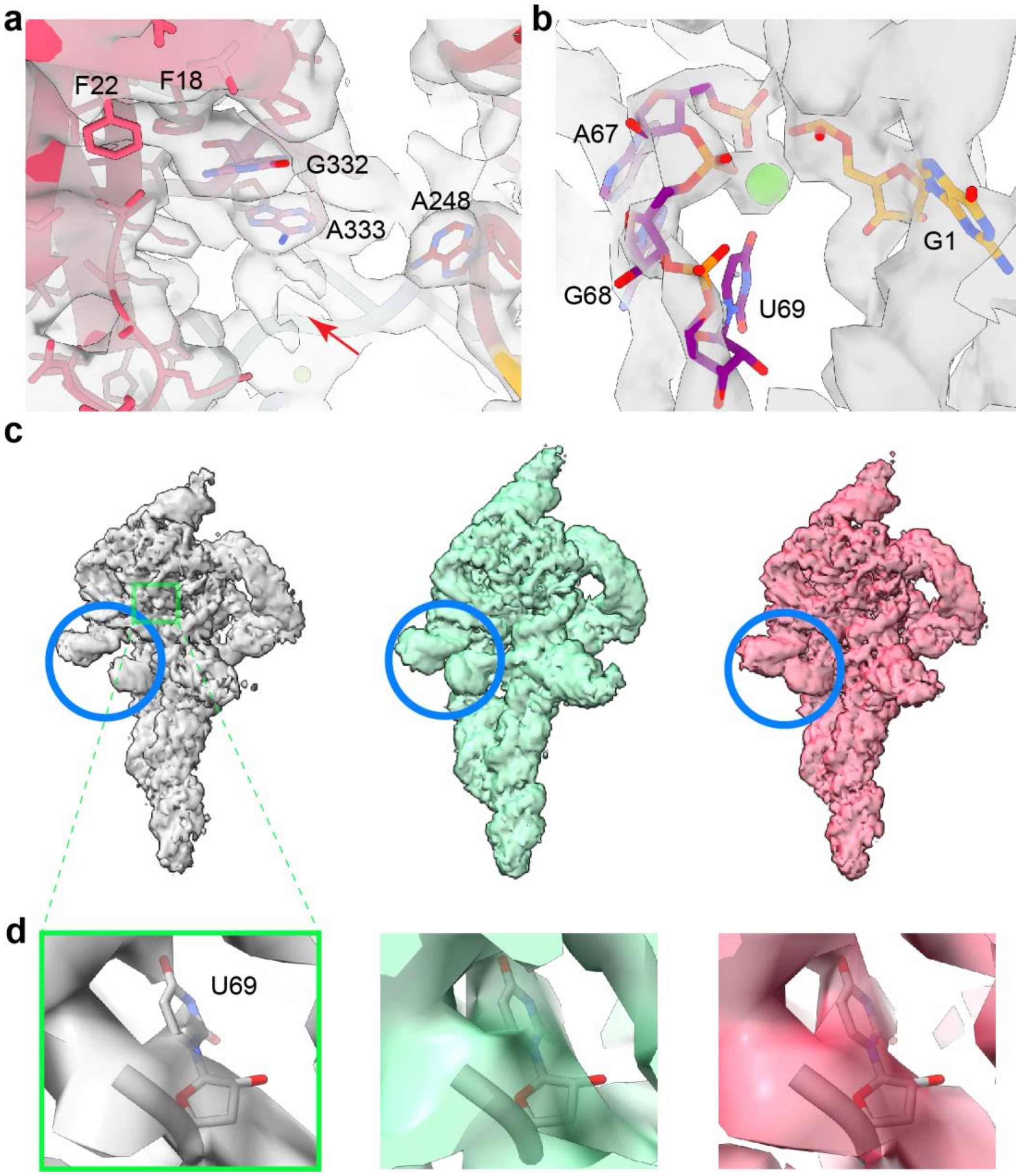

a, CryoEM density of the active site in RNase P holoenzyme. Full densities for A248, A333 and G332 are observed in the cryoEM map. In the holoenzyme, G332 stacks with F18 of rnpA and there is no connection in the cryoEM map between G332 and A333. Without 5' leader sequence bound, no map features for side-chain was observed for F22 presumably due to solvent exposure of the hydrophobic residue. A cloud of unassigned cryoEM density is observed below A333. **b,** CryoEM density around M1 ion and phosphate backbone that coordinate M1 ion and U69 nucleotide of RNase P. **c,** Three conformational classes from the AU_ES* particle set reveal distinct P1-L9 distances observed in the different states. **d,** Those classes with closer contacts between P1 and L9 (class 2, green; class 3, pink) also display

stronger U69 map features compared to class 1 (grey) with a greater distance between P1 and L9.

Supplementary Table 1 | CryoEM data collection, processing and model refinement statistics for *E. coli* RNase P holoenzyme and ES* complexes

	**Holoenzyme with Mg^{2+} (class 1)**	**Holoenzyme with Mg^{2+} (class 2)**	**RNase P with AU substrate**	**RNase P with GG substrate**
Microscope	Titan Krios	Titan Krios	Titan Krios	Titan Krios
Voltage (keV)	300	300	300	300
Image filter	BioQuantum	BioQuantum	BioQuantum	BioQuantum
Slit width (eV)	20	20	20	20
Super-resolution Pixel size (Å)	0.4363	0.4363	0.529	0.529
Symmetry	C1	C1	C1	C1
Defocus range (µm)	-0.5 to -2.5	-0.5 to -2.5	-0.5 to -2.5	-0.5 to -2.5
Electron dose ($e^-/Å^2$)	40	40	53	53
Micrographs	8497	8497	13,593	14,530
Number of particles	576,371	538,466	704,493	546,283
Map resolution at 0.143 FSC (Å)	3.1	3.1	3.2	3.4
B-factor	89.5	35.7	106	151.3
Model Refinement				
Atom	8,957	6,733	10,694	10,698
Residues	Protein: 112, Nucleotides: 373	Protein: 112, Nucleotide: 266	Protein: 112, Nucleotide:455	Protein: 112, Nucleotide: 455
CCmask	0.84	0.86	0.84	0.84
Resolution (FSC map vs. model at 0.5) (Å)	3.5	3.4	3.5	3.5
r.m.s deviations				
Bond lengths (Å)	0.005	0.002	0.006	0.007
Bond angles (°)	0.842	0.800	1.239	1.333
Clash score	25.47	10.68	32.53	42.28
MolProbity score	2.53	1.79	2.61	2.76
EMDB ID	26640	26638	26637	26636
PDB ID	7UO5	7UO2	7UO1	7UO0

Supplementary Figure 1a uncropped

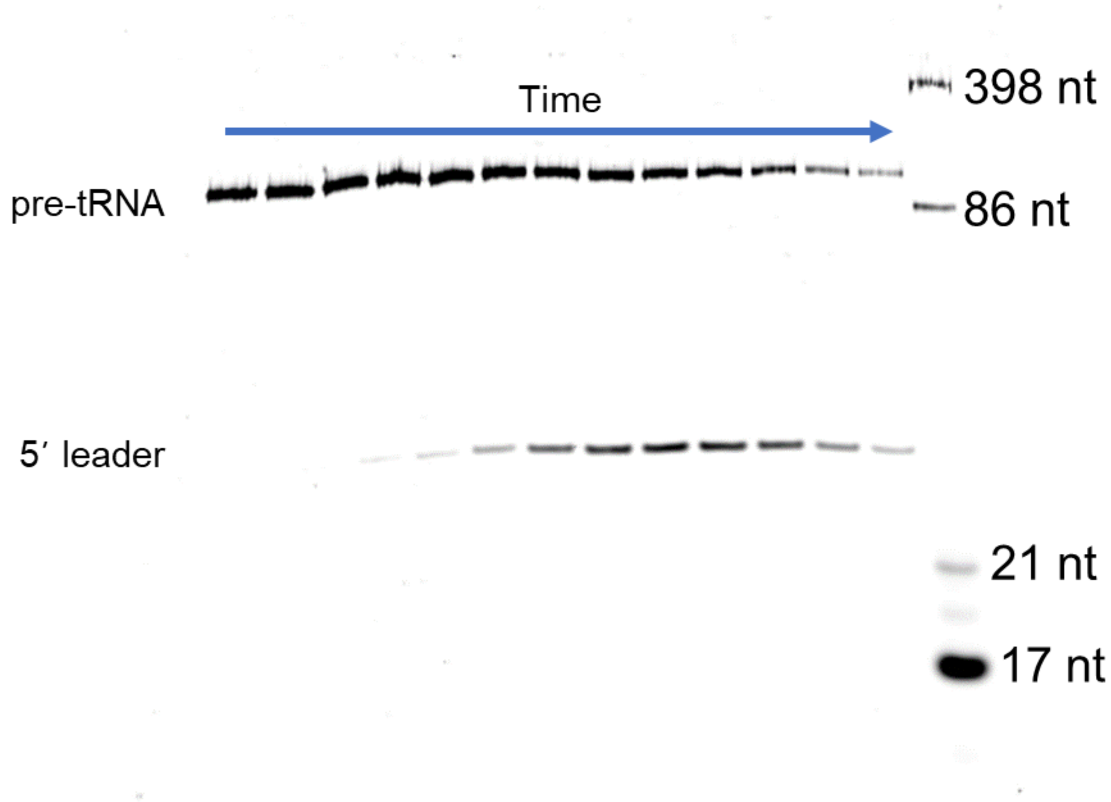

Supplementary Figure 4c uncropped

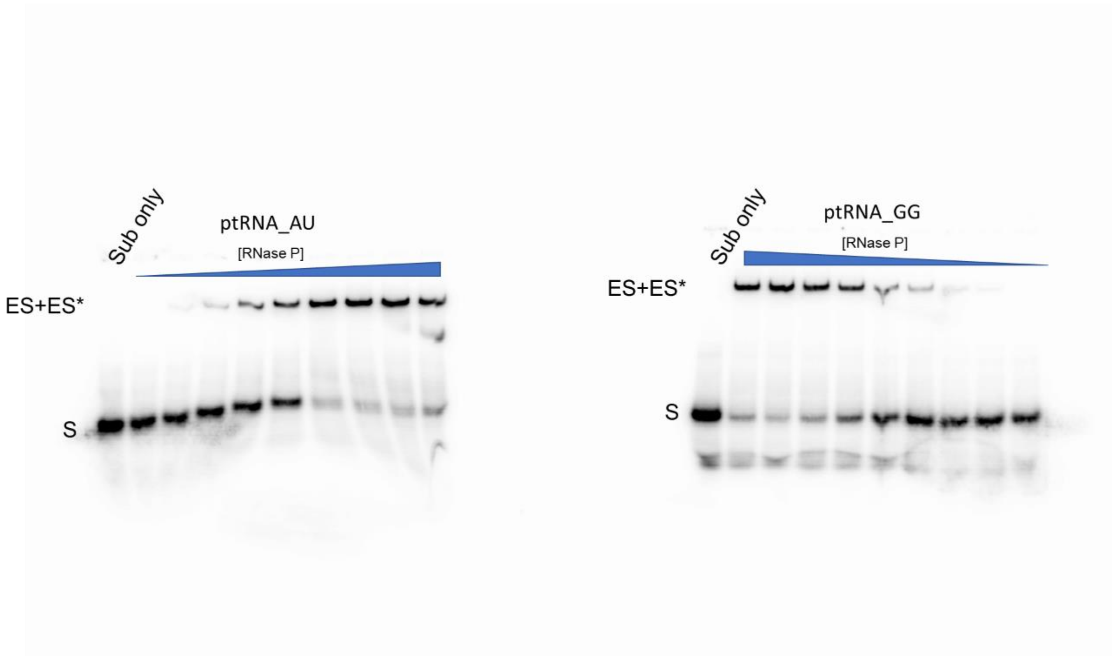

Supplement: Supplementary file 1 — Supplementary Information [file 41467_2022_32843_MOESM1_ESM.pdf]
