# Supplementary material for: Structural and mechanistic basis for recognition of alternative tRNA precursor substrates by bacterial ribonuclease P
Source: Nat Commun. 2022 Aug 31;13:5120. doi: 10.1038/s41467-022-32843-7 (PMC9433436; doi:10.1038/s41467-022-32843-7)
Supplement: Supplementary file 2 — Description of Additional Supplementary Files [file 41467_2022_32843_MOESM2_ESM.pdf]

File name: Supplementary Movie 1

Description: 3D variability analysis of RNase P holoenzyme (final particle set for class 1) with AU_ES* complex structure model fitting in the map.

File name: Supplementary Movie 2

Description: 3D variability analysis of AU_ES* final particle set with AU_ES* complex structure model fitting in the map.

File name: Supplementary Movie 3

Description: Morphing of structure models for RNase P holoenzyme and AU_ES* complex structure.
