# Supplementary material for: Structural and mechanistic basis for recognition of alternative tRNA precursor substrates by bacterial ribonuclease P
Source: Nat Commun. 2022 Aug 31;13:5120. doi: 10.1038/s41467-022-32843-7 (PMC9433436; doi:10.1038/s41467-022-32843-7)
Supplement: Supplementary file 6 — Reporting Summary [file 41467_2022_32843_MOESM6_ESM.pdf]

nature portfolio

Corresponding author(s): Michael E. Harris, Derek J. Taylor

Last updated by author(s): Apr 12, 2022

Reporting Summary

Nature Portfolio wishes to improve the reproducibility of the work that we publish. This form provides structure for consistency and transparency in reporting. For further information on Nature Portfolio policies, see our Editorial Policies and the Editorial Policy Checklist.

Statistics

For all statistical analyses, confirm that the following items are present in the figure legend, table legend, main text, or Methods section.

n/a	Confirmed	
☐	☒	The exact sample size (*n*) for each experimental group/condition, given as a discrete number and unit of measurement
☐	☒	A statement on whether measurements were taken from distinct samples or whether the same sample was measured repeatedly
☒	☐	The statistical test(s) used AND whether they are one- or two-sided<br>*Only common tests should be described solely by name; describe more complex techniques in the Methods section.*
☒	☐	A description of all covariates tested
☐	☒	A description of any assumptions or corrections, such as tests of normality and adjustment for multiple comparisons
☒	☐	A full description of the statistical parameters including central tendency (e.g. means) or other basic estimates (e.g. regression coefficient) AND variation (e.g. standard deviation) or associated estimates of uncertainty (e.g. confidence intervals)
☒	☐	For null hypothesis testing, the test statistic (e.g. *F*, *t*, *r*) with confidence intervals, effect sizes, degrees of freedom and *P* value noted<br>*Give P values as exact values whenever suitable.*
☒	☐	For Bayesian analysis, information on the choice of priors and Markov chain Monte Carlo settings
☒	☐	For hierarchical and complex designs, identification of the appropriate level for tests and full reporting of outcomes
☒	☐	Estimates of effect sizes (e.g. Cohen's *d*, Pearson's *r*), indicating how they were calculated

Our web collection on statistics for biologists contains articles on many of the points above.

Software and code

Policy information about availability of computer code

Data collection	Phosphorimager data was collected and analyzed using ImageQuant 4 (GE); cryoEM data was collected using Latitude S (at NCEF) and Leginon (at NCCAT). More detailed information can be found in the Method.
Data analysis	Origin 8.5 (OriginLab) and Excel (MS) were used for data analysis and graphing; atomic models for figures were generated using ChimeraX; cryoEM data was analyzed using CryoSparc 3.2.0, initial models were created and refined using Coot version 0.9.6 and MC-sym (https://major.iric.ca/MC-Sym/); the initial model for E.coli P protein was generated using AlphaFold II; models were refined into the cryoEM map using Phenix version dev-3951; structure models are validated using MolProbility 4.5.1; cryoEM density maps coloured by local resolution were generated using RELION 3.1. More detailed information can be found in the method.

For manuscripts utilizing custom algorithms or software that are central to the research but not yet described in published literature, software must be made available to editors and reviewers. We strongly encourage code deposition in a community repository (e.g. GitHub). See the Nature Portfolio guidelines for submitting code & software for further information.

Data

Policy information about availability of data

All manuscripts must include a data availability statement. This statement should provide the following information, where applicable:

- Accession codes, unique identifiers, or web links for publicly available datasets
- A description of any restrictions on data availability
- For clinical datasets or third party data, please ensure that the statement adheres to our policy

The cryoEM maps have been deposited in the Electron Microscope Data Bank under accession code EMD-26640 (RNase P holo class 1), EMD-26638 (RNaseP holo class 2), EMD-26637 (RNaseP with ptRNA_AU) and EMD-26636 (RNaseP with ptRNA_GG). The atomic coordinates for the deposited map have been deposited in the

Protein Data Bank under accession code 7UO5 (RNase P holo class 1), 7UO2 (RNase P holo class 2), 7UO1 (RNaseP with ptRNA_AU) and 7UO0 (RNaseP with ptRNA_GG). All maps and models will be released with publication.

Field-specific reporting

Please select the one below that is the best fit for your research. If you are not sure, read the appropriate sections before making your selection.

☒ Life sciences ☐ Behavioural & social sciences ☐ Ecological, evolutionary & environmental sciences

For a reference copy of the document with all sections, see nature.com/documents/nr-reporting-summary-flat.pdf

Life sciences study design

All studies must disclose on these points even when the disclosure is negative.

Sample size	*Describe how sample size was determined, detailing any statistical methods used to predetermine sample size OR if no sample-size calculation was performed, describe how sample sizes were chosen and provide a rationale for why these sample sizes are sufficient.*
Data exclusions	The pre-established criteria for exclusion of kinetic and thermodynamic data are to include all measurements in which the error of the values obtained from fitting of the primary data is less than 30%. All experimental replicates fell within these criteria.
Replication	Kinetic and thermodynamic experiments were repeated three or more times to permit calculation of standard deviations. The HTS-KIN experiments included two independent technical replicates from two independently generated randomized substrate populations.
Randomization	No samples were allocated into different experimental groups for assessment of alternative approaches, treatments, or experimental analysis. So this aspect of study design is not relevant.
Blinding	Blinding is not directly relevant, however the HTS-KIN data analysis for determination of relative rate constants were performed by two authors independently.

Reporting for specific materials, systems and methods

We require information from authors about some types of materials, experimental systems and methods used in many studies. Here, indicate whether each material, system or method listed is relevant to your study. If you are not sure if a list item applies to your research, read the appropriate section before selecting a response.

Materials & experimental systems

n/a	Involved in the study	
☒	☐	Antibodies
☒	☐	Eukaryotic cell lines
☒	☐	Palaeontology and archaeology
☒	☐	Animals and other organisms
☒	☐	Human research participants
☒	☐	Clinical data
☒	☐	Dual use research of concern

Methods

n/a	Involved in the study	
☒	☐	ChIP-seq
☒	☐	Flow cytometry
☒	☐	MRI-based neuroimaging

March 2021
